# Structural Basis of Cysteine Ligase MshC Inhibition by Cysteinyl-Sulfonamides

**DOI:** 10.3390/ijms232315095

**Published:** 2022-12-01

**Authors:** Luping Pang, Stijn Lenders, Evgenii M. Osipov, Stephen D. Weeks, Jef Rozenski, Tatiana Piller, Davie Cappoen, Sergei V. Strelkov, Arthur Van Aerschot

**Affiliations:** 1 Research Center of Basic Medicine, Academy of Medical Sciences, College of Medicine, Zhengzhou University, Zhengzhou 450001, China; 2 Biocrystallography, Department of Pharmaceutical and Pharmacological Sciences, KU Leuven, Herestraat 49, P.O. Box 822, 3000 Leuven, Belgium; 3 Medicinal Chemistry, Rega Institute for Medical Research, Department of Pharmaceutical and Pharmacological Sciences, KU Leuven, Herestraat 49, P.O. Box 1030, 3000 Leuven, Belgium; 4 Pledge Therapeutics, Gaston Geenslaan 1, 3001 Leuven, Belgium; 5 Laboratory of Microbiology, Parasitology and Hygiene, Department of Pharmaceutical Sciences, University of Antwerp, Universiteitsplein 1, 2610 Wilrijk, Belgium

**Keywords:** MshC, bi-substrate competitive inhibitors, protein-ligand co-crystal structures, structure-activity relationship, enzyme inhibition, *Mycobacterium tuberculosis*

## Abstract

Mycothiol (MSH), the major cellular thiol in *Mycobacterium tuberculosis* (Mtb), plays an essential role in the resistance of Mtb to various antibiotics and oxidative stresses. MshC catalyzes the ATP-dependent ligation of 1-*O*-(2-amino-2-deoxy-α-d-glucopyranosyl)-d-*myo*-inositol (GlcN-Ins) with l-cysteine (l-Cys) to form l-Cys-GlcN-Ins, the penultimate step in MSH biosynthesis. The inhibition of MshC is lethal to Mtb. In the present study, five new cysteinyl-sulfonamides were synthesized, and their binding affinity with MshC was evaluated using a thermal shift assay. Two of them bind the target with EC_50_ values of 219 and 231 µM. Crystal structures of full-length MshC in complex with these two compounds showed that they were bound in the catalytic site of MshC, inducing dramatic conformational changes of the catalytic site compared to the apo form. In particular, the observed closure of the KMSKS loop was not detected in the published cysteinyl-sulfamoyl adenosine-bound structure, the latter likely due to trypsin treatment. Despite the confirmed binding to MshC, the compounds did not suppress Mtb culture growth, which might be explained by the lack of adequate cellular uptake. Taken together, these novel cysteinyl-sulfonamide MshC inhibitors and newly reported full-length apo and ligand-bound MshC structures provide a promising starting point for the further development of novel anti-tubercular drugs targeting MshC.

## 1. Introduction

Tuberculosis (TB), caused by the Gram-positive pathogen *Mycobacterium tuberculosis* (Mtb), has existed for centuries and remains a major global public health threat. In 2018, 1.5 million TB deaths and 7 million new TB cases were identified by the World Health Organization (WHO) [1]. With the emergence and spread of multidrug-resistant (MDR) and extensively drug-resistant (XDR) Mtb strains, the efficiency of current anti-TB chemotherapeutics is consistently undermined, and there is an urgent and continuous demand for discovering new drugs [2,3]. Therefore, inhibiting enzymes involved in the important cellular processes of Mtb, in particular protein synthesis and antibiotic detoxification processes, is crucial to avert further complications from TB.

**Figure 1:**
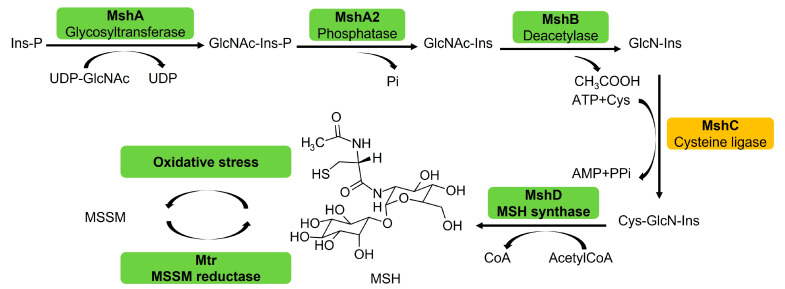
Biosynthetic and recycling pathway of MSH. MSH glycosyltransferase (MshA) links 1L-myo-inositol-1-phosphate (Ins-P) to N-acetylglucosamine (GlcNAc). MSH phosphatase (MshA2) produces GlcNAc-Ins. MSH deacetylase (MshB) generates 1-*O*-(2-amino-2-deoxy-α-d-glucopyranosyl)-d-*myo*-inositol (GlcN-Ins). MshC then links l-Cys with GlcN-Ins, and MSH synthase (MshD) acetylates l-Cys-GlcN-Ins to produce the final product, MSH. MSH autoxidation forms MSSM, which is reduced by MSH disulfide reductase (Mtr).

Like many actinomycetes, Mtb produces mycothiol (MSH), the functional equivalent of glutathione (GSH) in eukaryotes and most eubacteria, as the predominant low-molecular-weight thiol to protect against oxidative stress, electrophilic toxins and antibiotics [4,5,6]. This suggests that enzymes involved in the MSH biosynthesis and metabolism pathway may be potential targets for the development of selective antimycobacterial agents. MSH is synthesized through a series of complex enzyme-catalyzed reactions (Figure 1), in which the cysteine ligase MshC is the penultimate enzyme catalyzing the ATP-dependent condensation of cysteine and the GlcN-Ins intermediate. MshC has been proven to be essential for the maintenance of redox balance and metabolic homeostasis of Mtb [7,8]. Disruption or high-density mutagenesis in the *mshC* gene has been shown to be lethal for the in vitro growth of Mtb [9,10]. MshC, therefore, is considered as a potential and promising target for developing drugs to treat TB.

Being essential and specific to the lifecycle of Mtb, compounds targeting MshC, in theory, would have a limited chance of hitting off-targets within the host or host microbiome, reducing the possibility of undesired toxicity. One exception could be the host cysteinyl-tRNA synthetase (Hs-CysRS)—and, to our advantage, Mtb-CysRS—as the overall fold of the MshC catalytic domain strongly resembles the one for CysRSs [11,12]. This is the result of both enzymes catalyzing the same cysteinyl-adenosine (Cys-AMP) intermediate formation before transferring the activated cysteine to different substrates (GlcN-Ins and tRNA^Cys^ for MshC and CysRS, respectively). Thus far, there is no specific drug available for the inhibition of MshC. Screening of chemical libraries afforded NTF1836 [13] and dequalinium [14] chloride, both exhibiting moderate in vitro MshC inhibitory activity and Mtb growth inhibition. However, both of them were proved to be cytotoxic to mammalian cells and hence were not pursued further. Attempts to exploit these small molecule scaffolds or identify new chemical entities have also been hampered by the lack of the full-length molecular structure of MshC.

**Figure 2:**
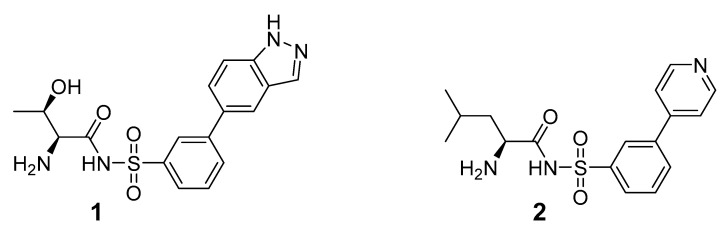
Respective lead compounds described for inhibition of bacterial ThrRS (developed by Trius Pharmaceuticals) and LeuRS (developed by Oxford Drug Design), respectively.

Structurally, MshC is similar to class I aminoacyl-tRNA synthetases (aaRSs), especially to CysRS, as mentioned above. AaRSs catalyze the ligation of amino acids with their cognate tRNA in an ATP-dependent manner. These enzymes are divided into two classes based on two different folds of the catalytic core [15,16]. Class I aaRSs share a similar Rossmann fold (belonging to the HUP superfamily) and have two conserved HIGH and KMSKS signature sequence motifs, while the catalytic domain of class II aaRSs adopts a six-stranded β sheet [15,16]. The aaRSs families have been pursued as viable targets for the discovery and design of new antibiotics for a number of decades [17,18,19,20,21,22,23]. Herein, aminoacyl-sulfamoyl adenosines (aaSAs) are well-known, high-affinity ligands of aaRS, effectively mimicking the natural aminoacyl-adenosine (aa-AMP) intermediate in which the non-hydrolysable sulfamoyl-moiety mimics the binding and function of the phosphoryl group of aa-AMP. However, these compounds lack species selectivity and are devoid of antibacterial properties due to their highly polar nature. Recently, “Trius pharmaceuticals” discovered ThrRS-targeting *m*-substituted aromatic sulfonamides (Figure 2) [24]. These substituted aryl sulfonamides mirror the sulfamoyl adenosine part of the threonyl-SA [24]. Analogous success was achieved by Oxford Drug Design targeting LeuRS [25]. Both series of compounds showed very potent enzymatic inhibitory and antibacterial activity as well as species selectivity.

Given these advances, we designed and synthesized a small series of cysteinyl-sulfonamide compounds which were based on the same scaffold and, therefore, potentially capable of simultaneously targeting CysRS and MshC. Using the thermal shift assay, two compounds were confirmed to bind to MshC. Subsequently, the co-crystal structures of these compounds bound to full-length MshC were determined. This work elucidates how this sulfonamide scaffold works with the class I aaRS-like enzyme and provides a starting point for the further design and optimization of anti-tubercular drugs targeting these enzymes.

## 2. Results and Discussion

### 2.1. Design and Synthesis of Cysteinyl-Sulfonamides

**Figure 3:**
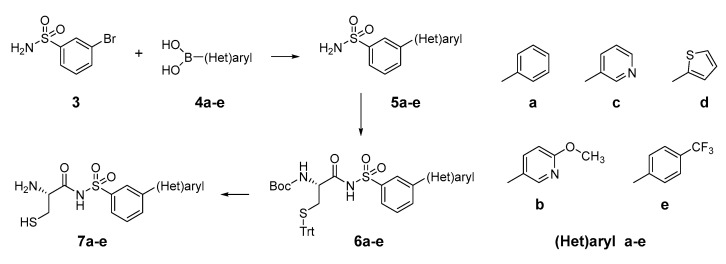
Synthetic scheme for the cysteinyl sulfonamide congeners **7a**–**e**.

Based on the previously published heterocyclic sulfonamide congeners targeting ThrRS or LeuRS (Figure 2), we designed and synthesized a small series of cysteinyl-sulfonamide compounds for the potential simultaneous targeting of CysRS and MshC (Figure 3). Starting from 3-bromophenylsulfonamide (**3**), a Suzuki reaction with arylboronic acids **4a**–**e** afforded the sulfonamides **5a**–**e** in moderate yields. The latter were acylated with protected l-cysteine, followed by deprotection to afford the target cysteinyl sulfonamides **7a**–**e**.

### 2.2. Binding Affinity of Compounds against M. smegmatis MshC

The binding affinity of these compounds with *M. smegmatis* MshC (Ms-MshC), a homologue to MshC from *M. tuberculosis* (Mtb-MshC), was determined by a thermal shift assay (TSA). We here opted for Ms-MshC to carry out affinity determination as the enzyme highly resembles Mtb-MshC with an overall sequence identity and similarity of 79% and 88%, respectively. Further examination of the active site of these two proteins, which are, in their first step, responsible for the cysteinyl-adenosine formation, showed 93% identity and 97% similarity. Therefore, the binding affinity of the synthesized compounds evaluated against Ms-MshC appears to likewise reflect their potential binding with Mtb-MshC. In addition, the availability of Ms-MshC is beneficial for the following structural studies.

In principle, compound binding usually improves the thermal stability of the protein, which then leads to an increase in the melting temperature (T_m_) during the denaturation process. Furthermore, when a specific compound induces a bigger increase in T_m_, this compound usually possesses a higher binding affinity compared to other ligands sharing a similar scaffold [26]. To eliminate the effect of the solvent in this experiment, ∆T_m_ was calculated as the difference between T_m_ of Ms-MshC with and without 10% (*v*/*v*) either dimethyl sulfoxide (DMSO) or ethylene glycol (EDO). DMSO clearly lowers the thermal stability of the protein by decreasing the T_m_ of Ms-MshC by 3.38 °C, while EDO decreases the T_m_ by only 0.48 °C (Table 1). Therefore, EDO was selected as the solvent to assist in dissolving the respective compounds in the subsequent experiments.

The natural substrate l-Cys and ATP solely increased the T_m_ of Ms-MshC by 3.09 and 1.35 °C at 10 mM concentration, respectively, but almost did not change T_m_ at 1 mM (Table 1). In contrast, when l-Cys and ATP were simultaneously added both at 100 μM or 1 mM concentration in the presence of 5 mM MgCl_2_, significant increases of ∆T_m_ of 3.76 and 8.08 °C, respectively, were observed. This suggests that the formation of the Cys-AMP reaction intermediate is catalyzed by the enzyme in this case, in line with its higher binding affinity compared to single substrates. Subsequently, 100 μM and 1 mM concentrations of each synthesized compound were applied to measure their effects on the T_m_ of Ms-MshC in the presence of EDO. However, only compound **7d** showed a 0.99 °C increase in T_m,_ while the others had no effect (Table 1).

**Figure 4:**
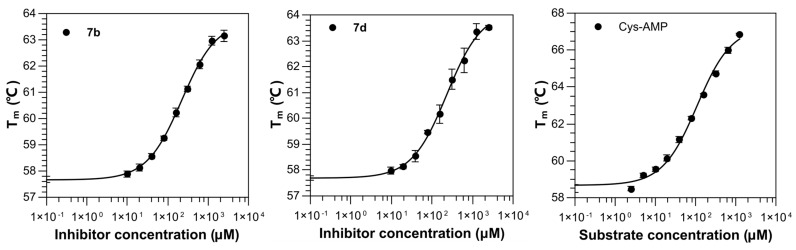
Titration curves of compounds measured by thermal shift assay. The experiments were performed in triplicate with error bars shown.

The synthesized compounds contain a cysteinyl moiety which can be easily oxidized to yield the disulfide form, and these dimerized compounds may not fit into the active center of MshC. This may explain their surprisingly weak stabilization effects on protein. Therefore, to eliminate the effect of oxidation, a 1 mM reducing agent, tris-(2-carboxyethyl)phosphine (TCEP), was added to the evaluation system, and then the effects of the various compounds on the T_m_ of the protein were reassessed. In the absence of Ms-MshC ligands, TCEP had no effect on the thermal stability of the protein. In the following experiments, ∆T_m_ was calculated as the difference between T_m_ of Ms-MshC in the presence and absence of 100 µM and 1 mM, respectively, of each compound. The results demonstrated that compounds **7b** and **7d** cause an increase in the ∆T_m_ (1.24 and 1.10 °C at 100 μM; 3.08 and 3.75 °C at 1 mM), suggesting that the TCEP-reduced compounds are potential binders of Ms-MshC. Measurements of dilution series for these two compounds showed K_i_^app^ of 219 and 231 µM respectively, which is only 2-fold weaker binding compared with the natural intermediate Cys-AMP (K_i_^app^ of 107 µM) in this evaluation system (Table 1 and Figure 4).

A comparison of these five synthesized compounds showed that the heterocycle instead of a phenyl ring at C3 of the phenyl sulfonamide (compounds **7b**, **7c** and **7d**) is beneficial for binding. Despite both compounds **7b** and **7c** containing a pyridine moiety, the ortho-methoxy substituent on the pyridine further improved the binding affinity compared with the non-modified compound **7c**, suggesting the modification on the ortho-position of the pyridine ring seems more suitable for binding. Combined, these cysteinyl-sulfonamide-based compounds are potential new binders for Ms-MshC.

### 2.3. Structural Study on the Inhibitory Mechanism of Compounds ***7b*** and ***7d***

In the past, the first partial crystal structure of MshC from *M. smegmatis* complexed with cysteinyl adenylate analogue, 5′-*O*-[*N*-(l-cysteinyl)-sulfamonyl] adenosine (CSA), was determined. Here, the formed MshC-CSA was treated with trypsin prior to crystallization [27]. It has been reported that the overall tertiary structure of MshC is similar to that of CysRS, which catalyzed the formation of cysteine-charged tRNA. However, as a result of trypsin proteolysis, this structure lacks the KMSKS loop, which is normally situated near the substrate binding site. In CysRS, the KMSKS motif is responsible for the binding and positioning of the L-shaped tRNA molecule as well as amino acid activation. It was proposed that the homologous loop in MshC is involved in the formation of the adenylate intermediate in the first-half reaction and possibly also important for the binding of the GlcN-Ins substrate in the second-half reaction [27]. Therefore, a crystal structure of full-length MshC is required for a better understanding of the function of this enzyme and elucidation of the binding mechanism of MshC inhibitors.

**Figure 5:**
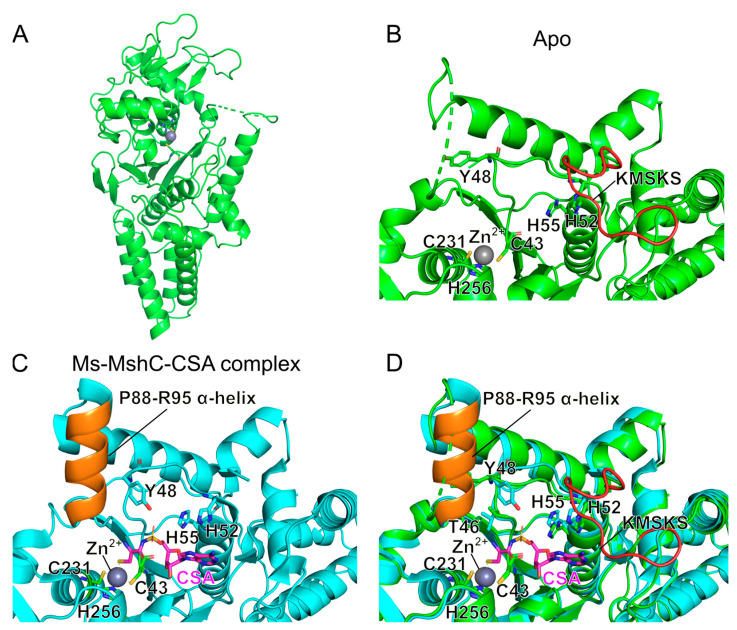
Comparison of the crystal structures of full-length and proteolyzed MshC from *M. smegmatis*. (**A**) Ligand-free structure of full-length MshC. (**B**) Zoom in of the catalytic domain of apo form MshC; (**C**) Zoom in of the catalytic domain of MshC in complex with CSA [27] (PDB ID: 3C8Z); (**D**) Structural superposition of apo form (green) and CSA-bound MshC (cyan). Protein backbones of both structures are shown as cartoon representations, while ligand and crucial protein residues are shown as sticks, and the co-factor Zn^2+^ ion is shown as a grey ball. The red- and orange-colored regions represent the KMSKS loop in the apo structure and the P88–R95 helix in the published CSA-bound structure, respectively.

Here, we determined the crystal structure of the full-length Ms-MshC at 2.4 Å resolution (Figure 5A and Table 2). While the published MshC structure in complex with CSA contains two macromolecules in the asymmetric unit (ASU), all structures solved in this work only possess one macromolecule per ASU despite crystalizing in two different space groups (Table 2).

In contrast to the published MshC-CSA complex (PDB ID: 3C8Z) that had been truncated through trypsin proteolysis, the newly obtained structure reveals the intact KMSKS loop. At the same time, the α-helix containing residues P88–R95 is disordered in our apo structure, while it is fully structured in the CSA-bound structure (Figure 5B,C). Detailed analysis of these two structures showed that CSA binding induces a shift of the T46-H52 loop region towards the active site, whereby the side chain of Y48 is flipped inwards into the binding pocket. This concerted conformational change avoids the steric clash between the side chain of Y48 in the apo structure and the ordered positioning of the P88–R95 containing α-helix upon CSA binding (Figure 5C,D).

**Figure 6:**
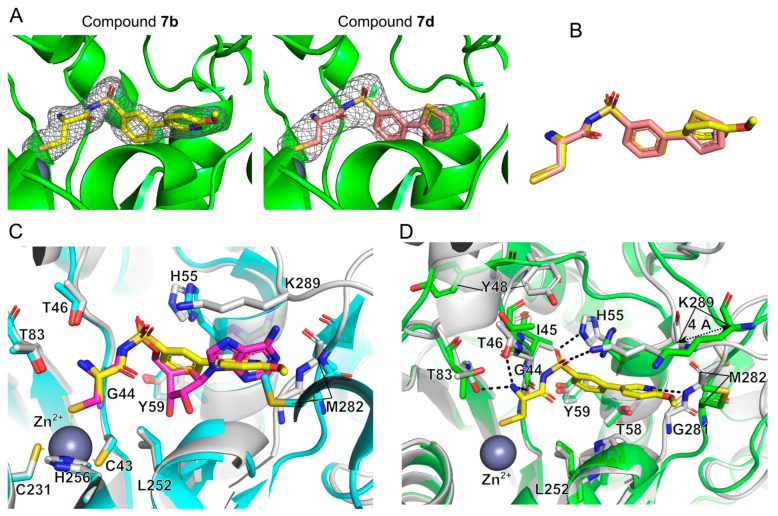
The binding mode of the sulfonamide-based compound with Ms-MshC. (**A**) The calculated electron density map (omit map) of compounds **7b** and **7d** in the catalytic site of Ms-MshC. Maps were determined in Phenix.Polder map [28] and countered at 3.5 σ. (**B**) Superposition of compounds **7b** and **7d** based on the alignment of protein backbones. (**C**) Structural superposition of CSA-bound (cyan) and compound **7b**-bound (grey) Ms-MshC. (**D**) Structural superposition of the apo form of MshC (green) and MshC in complex with compound **7b** (grey). Protein backbones were shown as cartoon representations, while ligands and essential protein residues were shown as sticks, with the co-factor Zn^2+^ ion shown as a grey sphere. H-bonds were shown as black dashed lines. The movement of ^289^KMSKS^293^ loop (~4 Å) induced by compound binding in Figure 6D was measured based on the different positioning of K289 C_α_ atom between these two structures.

To clarify the binding mechanism of the newly synthesized sulfonamide-based compounds, we also have co-crystallized Ms-MshC with compounds **7b** and **7d** and determined the structures of these complexes at 2 and 2.8 Å resolution, respectively (Table 2). The electron density map unambiguously showed both compounds binding in the active site in a very similar manner (Figure 6A,B). The superposition of both complexes reveals little change in the protein backbone (RMSD of 0.33 Å using coordinates of 370 Cα atoms). Therefore, detailed structural analysis was carried out only for the higher resolution complex involving the better binder **7b**.

In comparison with the structure of Ms-MshC in complex with CSA, the cysteinyl moiety of compound **7b** is fully superposed with the same chemical group of CSA (Figure 6C). The thiol group of the ligand and the side chains of C43, C231 and H256 coordinate the co-factor Zn^2+^ ion. The presence of Zn^2+^ is considered the major recognition mechanism of substrate l-Cys from the amino acids pool, as reported for MshC-homolog CysRS [12]. The α-amino group of the cysteinyl moiety forms three hydrogen bonds with the hydroxyl oxygen of side chains of T46 and T83 and the main-chain oxygen of G44, which is likely important for the correct chirality selection of this amino acid (Figure 6D). In addition, the binding of CSA or the cysteinyl-containing analog induces the same inward movement of the ^44^GITPY^48^ loop surrounding the back pocket of MshC. This results in the inward flipping of the side chain of Y48, which may prevent the hydrolysis of the cysteinyl-adenylate reaction intermediate. The presence of the sulfonamide group in compound **7b**, similar to the sulfamoyl group in CSA, induces the inward movement of the ^289^KMSKS^293^ loop with the largest C_α_ shift of 4 Å. This leads to the formation of one salt bridge between the sulfonamide oxygen and the amine group of the K289 residue of the ^289^KMSKS^293^ motif, and one H-bond between the sulfonamide oxygen and Nε of H55 from the HIGH motif, respectively. Since the sulfonamide and sulfamoyl groups in both structures (Figure 6D) are fully overlaid, it seems rational to hypothesize that the sulfamoyl group in CSA likely induces a similar conformational change of the KMSKS loop. However, due to the limited trypsinolysis treatment of the protein before crystallization with the CSA ligand, the KMSKS loop was likely cleaved. This may explain why the KMSKS loop could not be observed in the previously published CSA-bound structure of MshC.

The phenyl group of compound **7b** occupies the ribose binding pocket of MshC but only forms hydrophobic interactions with surrounding protein residues (Figure 6C,D). The substituted pyridine moiety is superposed with the adenine base of CSA located in the ATP binding cavity. Positioning of the pyridine plane is sterically clashing with the position of M282 in the CSA-bound structure, which forms hydrophobic interactions with the side chain of the adenine base. Therefore, in compound **7b**’s bound structure, the backbone peptide planes of G281-M282 and M282-I283 are flipped. On the one hand, this avoids the steric clash and accommodates the binding of pyridine, and on the other hand, it generates one H-bond between the nitrogen atom of the pyridine ring and the backbone NH of M282, thus increasing the binding affinity for this ligand (Figure 6D). The lack of an H-bond between the thiophene ring of **7d** and active site residues would suggest that a lower binding potency should be expected relative to compound **7b**. This is in good agreement with the TSA results (Table 1). Taken together, the sulfonamide-based compounds can probably replace the non-selective sulfamoyl-containing analog CSA, acting as a new class of Ms-MshC inhibitors.

### 2.4. Anti-Mycobacterium Activity of the Compounds

The synthesized compounds were subsequently profiled for antimycobacterial activity in a whole-cell screening assay. The panel of test organisms included the *Mycobacterium tuberculosis* H37Ra lab strain, the *Δmtr::Hyg* derivative strain and *Mycobacterium abscessus* containing PSMT-1. The *Mtb* H37Ra *Δmtr::Hyg* strain carries a deletion in the Rv2855 gene encoding mycothiol reductase (Mtr) (Table 3). Mtr itself is the key enzyme to reduce oxidized mycothione to mycothiol, thereby maintaining the reductive intracellular environment within the bacillus. In addition, the compounds have been evaluated against *M. abscessus*, the causative agent of opportunistic nontuberculous infections in immune-compromised persons. However, based on luminescence, no activity could be observed for the test compounds for concentrations up to 100 µM (Table 3). One possible explanation for the failure of target engagement of the compounds in the whole-cell evaluation is to attribute the lack of potency to poor cellular pharmacodynamics or pharmacokinetics of the compounds. Moreover, since compounds **7b** and **7d** are Cys-AMP competitive inhibitors, but as their EC_50_ values are two-fold lower than the reaction intermediate, it would be challenging for the synthesized compounds to efficiently block the catalytic activity of MshC inside the cell. This could be another explanation for their weak antimycobacterial activities. Therefore, further work should look for higher affinity binders based on the **7b**-bound MshC structure. Using docking and molecular dynamics simulations, various heterocycles substituting for the pyridine ring hereto could be evaluated in an effort to rationally optimize these scaffold compounds. This should pave the way for the development of new MshC antimycobacterial drug candidates.

## 3. Materials and Methods

### 3.1. Reagents and Analytical Procedures

Reagents and solvents were purchased from commercial suppliers and used as provided and previously described [29]. ^1^H and ^13^C NMR spectra of the compounds were recorded on a Bruker UltraShield Avance 300 MHz (Brüker, Fällanden, Switzerland) or, when needed, on a 500 MHz and 600 MHz spectrometer. High-resolution mass spectra were recorded on a quadrupole time-of-flight mass spectrometer (SYNAPT G2 HDMS, Waters, Milford, MA, USA) equipped with a standard ESI interface; all these analytical techniques were used as described previously [29]. All the ^1^H NMR, ^13^C NMR and MS spectra data were summarized in Supplementary File S1.

### 3.2. Chemical Synthesis and Analysis

#### 3.2.1. Synthesis of Compounds **5a**–**e**: General Procedure A

3-Bromobenzenesulfonamide (**3**) (300 mg, 1.26 mmoL, 1 eq) and the respective boronic acids **4a**–**e** (1.4 mmoL, 1.1 eq) and K_2_CO_3_ (538 mg, 3.9 mmoL, 3.1 eq) were dissolved in 15 mL of a 4:1 mixture of 1,4-dioxane:water. Pd(dppf)(Cl)_2_ (175 mg, 0.25 mmoL, 0.2 eq) was added, and the reaction mixture was stirred overnight at 110 °C. After consumption of the starting material, the reaction was cooled down to room temperature and diluted with 30 mL of MeOH. The mixture was filtered over a celite plug and dried using anhydrous Na_2_SO_4_. The mixture was filtered again, and the solvents were evaporated to dryness. The resulting crude was purified using silica gel chromatography to obtain **5a**–**e**.

#### 3.2.2. Synthesis of Compounds **6a**–**e**: General Procedure B

The respective sulfonamide **5a**–**e** (1 eq), *N*-(tert-butoxycarbonyl)-S-trityl-l-cysteine (1.2 eq), O-Benzotriazole-N,N,N′,N′-tetramethyluronium-hexafluoro-phosphate (HBTU, 1.5 eq) and triethylamine (3 eq) were dissolved in 4 mL of N,N-dimethylformamide (DMF) and stirred overnight. After thin layer chromatography (TLC) showed full consumption of the starting material, the reaction mixture was diluted with 25 mL of ethyl acetate (EtOAc) and washed with 3 × 25 mL of brine. The organic phase was dried using anhydrous Na_2_SO_4_, filtered and evaporated to dryness. The resulting crude was purified using silica gel chromatography affording **6a**–**e**.

#### 3.2.3. Synthesis of Compounds **7a**–**e**: General Procedure C

An aliquot of 1 mL of trifluoroacetic acid (TFA) was added to a solution of the respective starting material (**6a**–**e**) in 4 mL of dichloromethane (DCM). Triethylsilane (2.5 eq) was added, and the solution was stirred for 1 h. The reaction was quenched with saturated NaHCO_3_ and extracted using DCM. The organic phases were collected, dried using anhydrous Na_2_SO_4_ and filtered. The organic phase was evaporated to dryness, and the resulting crude was purified using silica gel chromatography, affording the final sulfonamides **7a**–**e**.

*N-([1,1′-biphenyl]-3-ylsulfonyl)-2(R)-amino-3-mercaptopropanamide* (**7a**). ^1^H NMR (300 MHz, (MeOD): δ (ppm) = 8.12 (s, 1H), 7.99 (bs, 2H, NH), 7.85 (d, *J* = 7.53 Hz, 2H), 7.68 (s, 1H), 7.66 (s, 1H), 7.60 (t, *J* = 7.06 Hz, 1H), 7.51 (t, *J* = 7.29 Hz, 2H), 7.43 (d, *J* = 7.53 Hz, 1H), 3.83 (s, 1H), 2.91 (s, 2H); ^13^C NMR (75 MHz, (MeOD): δ (ppm) = 129.23, 129.16, 129.10, 128.42, 128.13, 126.85, 126.32, 126.19, 125.53, 55.64, 25.21; HRMS (ESI): calcd. for C_15_H_17_N_2_O_3_S_2_ [M+H]^+^: 337.0675, found: 337.0678.

*2(R)-Amino-3-mercapto-N-((3-(6-methoxypyridin-3-yl)phenyl)sulfonyl)propenamide* (**7b**). ^1^H NMR (300 MHz, (MeOD): δ (ppm) = 8.50 (d, *J* = 2.22 Hz, 1H), 8.11–7.98 (m, 4H), 7.92–7.83 (m, 2H), 7.63 (t, *J* = 8.32 Hz, 1H), 6.95 (d, *J* = 7.21, 1H), 3.91 (s, 3H), 3.79 (s, 1H), 2.93–2.84 (m, 2H); ^13^C NMR (75 MHz, (MeOD): δ (ppm) = 163,.53, 145.02, 137.73, 137.40, 129.37, 126.14, 124.96, 110.91, 53.47, 40.13, 30.40; HRMS (ESI): calcd. for C_15_H_18_N_3_O_4_S_2_ [M+H]^+^: 368.0733, found: 368.0730.

*2(R)-Amino-3-mercapto-N-((3-(pyridin-3-yl)phenyl)sulfonyl)propanamide* (**7c**). ^1^H NMR (300 MHz, (DMSO): δ (ppm) = 8.97 (s, 1H), 8.70 (d, *J* = 4.54 Hz, 1H), 8.25 (d, *J* = 8.17 Hz, 1H, 8.18 (s, 1H), 8.05 (bs, 2H, NH), 7.97 (t, *J* = 8.17 Hz, 2H), 7.74–7.62 (m, 2H), 2.93 (s, 2H); ^13^C NMR (75 MHz, (DMSO): δ (ppm) = 168.62, 140.56, 135.82, 130.43, 129.60, 129.00, 128.23, 126.69, 126.19, 125.04, 122.54, 55.90, 25.30.

*2(R)-Amino-3-mercapto-N-((3-(thiophen-2-yl)phenyl)sulfonyl)propanamide* (**7d**). ^1^H NMR (300 MHz, (DMSO): δ (ppm) = 8.13 (s, 1H), 8.05 (bs, 2H, NH), 7.98–7.89 (m, 2H), 7.80 (d, *J* = 8.26 Hz), 7.72–7.68 (m, 1H), 7.61–7.52 (m, 2H), 3.89 (s, 1H), 2.92 (d, *J* = 3.88 Hz, 2H); ^13^C NMR (75 MHz, (DMSO): δ (ppm) = 130.19, 129.65, 128.17, 126.47, 126.26, 125.09, 122.68, 55.84, 25.42; MS (ESI): calcd. for C_13_H_14_N_2_O_3_S_3_ [M+H]^+^: 343.0, found: 342.8.

*2(R)-Amino-3-mercapto-N-((4′-(trifluoromethyl)-[1,1′-biphenyl]-3-yl)sulfonyl)propanamide* (**7e**). ^1^H NMR (300 MHz, (DMSO): δ (ppm) = 8.17 (s, 1H), 8.00–7.83 (m, 8H), 7.64 (t, *J* = 7.30 Hz, 1H), 3.80 (s, 1H) 2.91 (s, 2H); ^13^C NMR (150 MHz, (DMSO): δ (ppm) = 143.86, 19.11, 128.43, 127.70, 126.32, 126.07, 126.04, 125.80, 55.83, 29.05; MS (ESI): calcd. for C_16_H_16_F_3_N_2_O_3_S_2_ [M+H]^+^: 405.1, found: 404.9.

### 3.3. Cloning, Expression and Protein Purification

The DNA sequence encoding full-length *M. smegmatis* MshC (Ms-MshC, UniProt accession ID: A0QZY0) was amplified by polymerase chain reaction (PCR) from genomic DNA isolated from *Mycobacterium smegmatis*. The amplified gene was separated by agarose gel electrophoresis and purified by a gel extraction kit (Qiagen, Hilden, Germany). The purified gene was subsequently cloned into the pETRUK vector, an in-house derivative of pETHSUL [30] that yields a fusion protein with a SUMO tag at the N-terminus for expression in *E. coli* Rosetta 2 (DE3) pLysS cells. Initial trials showed that the SUMO tag of SUMO-fused Ms-MshC was not able to be cleaved efficiently by SUMO hydrolase. Therefore, a glycine spacer was added between the C-terminal SUMO tag residue and the first methionine residue of Ms-MshC. Isolation of the protein was similar to that previously reported for LeuRS [29,31,32]. Briefly, following culture of transformed Rosetta 2 (DE3) pLysS ZYP-5052 auto-induction medium [33], cells were harvested by centrifuge and lysed by sonication in cation exchange buffer A (25 mM HEPES-NaOH pH 7, 150 mM NaCl and 5 mM β-mercaptoethanol) supplemented with 1 mM MgCl_2_ and 100 U cold-active Cryonase (Takara, Shiga, Japan). The lysis was clarified by centrifugation at 18,000× *g* for 45 min, and the resulting supernatant was applied onto a 5 mL Hitrap HP SP column (Cytiva, Marlborough, MA, USA). The protein was eluted by a linear gradient with Cation exchange buffer B (25 mM HEPES-NaOH pH 7, 1 M NaCl and 5 mM β-mercaptoethanol). Fractions corresponding to the SUMO-fused Ms-MshC were combined, followed by SUMO hydrolase treatment to remove the SUMO tag. This combined mixture was dialyzed in buffer containing 20 mM Tris-HCl pH 7, glycerol 10% (*w*/*v*) overnight at 4 °C to remove the salt and was then filtered by a 0.45 μm syringe filter (Millipore, Burlington, MA, USA) and loaded onto a Hitrap HP SP column (Cytiva, Marlborough, MA, USA) to remove the SUMO tag and SUMO hydrolase. The flow through containing Ms-MshC was further purified by anion exchange chromatography and size exclusion chromatography. Purified Ms-MshC was concentrated to 20 mg/mL in the final buffer (10 mM Tris-HCl pH 7, 100 mM NaCl and 2.5 mM β-mercaptoethanol) and stored at −80 °C.

### 3.4. Crystallization

The crystals of the apo form of Ms-MshC were obtained by the hanging drop vapor diffusion method at 20 °C. Briefly, 10 mg/mL protein in 10 mM Tris-HCl pH 7, 100 mM NaCl and 2.5 mM β-mercaptoethanol was mixed with the reservoir solution containing 25 mM CaCl_2_, 25 mM MgCl_2_, PEG8000 5–8% (*w*/*v*), Morpheus buffer system 2 pH 7.1, and ethylene glycol 20% (*v*/*v*) in a 1:1 ratio, which was then equilibrated over 1 mL of the same solution. Suitable crystals were harvested using a nylon cryo-loop. The crystals were briefly immersed in the reservoir solution supplemented with 22% (*v*/*v*) ethylene glycol as a cryoprotectant and then flash-frozen in liquid nitrogen for subsequent data collection.

For the MshC-ligand complex, the protein at a concentration of 10 mg/mL in 10 mM Tris-HCl pH 7, 100 mM NaCl and 2.5 mM β-mercaptoethanol was mixed with a final concentration of 1 mM compound and 1 mM tris(2-carboxyethyl)phosphine (TCEP), which was then incubated on ice for 1 h. Before crystallization experiments, the protein mixture was centrifuged at 12,000× *g* for 10 min at 4 °C. This cleared mixture was subjected to extensive crystallization screening against a broad series of commercially available screens utilizing the sitting drop vapor diffusion method at 20 °C. Crystals of MshC in complex with either compound **7b** or 7**d** were found in conditions from the Morpheus screen in dispensed droplets comprised of 300 nL protein mixture and 150 nL reservoir solution containing PEG550MME 20% (*v*/*v*), PEG20000 10% (*w*/*v*), Morpheus divalent 60 mM and Morpheus buffer system 3 pH 8.5. The crystals were cryoprotected by passage through paraffin oil and flash-frozen in liquid nitrogen prior to data collection.

### 3.5. Data Collection and Structure Determination

X-ray diffraction data were collected at 100 K on a Beamline ID23-1 (ESRF, Grenoble, France) and Proxima I (Soleil, Paris, France) using a standard data collection setup. Data were processed using the autoPROC package [34]. The initial structure solution was determined by molecular replacement using Phaser [35] employing a modified model of Ms-MshC (PDB code: 3C8Z), whereby the bound ligand was removed as a starting search model. The structures were refined by alternating the steps of the manual building method in COOT [36] and refinement with Phenix.refine [28]. The final structures were qualified using the validation tools available on the Protein Data Bank server [37]. The corresponding data collection and refinement statistics are summarized in Table 2. Structural figures were prepared with Pymol (version 2.0.4).

### 3.6. Thermal Shift Assay

The binding affinity of the synthesized compounds to *M. smegmatis* MshC (Ms-MshC) was evaluated by applying a fluorescence-based thermal shift assay (TSA). A 20 μL reaction system containing 0.2 mg/mL protein, 1× thermal shift dye (ThermoFisher Scientific, Waltham, MA, USA), 50 mM HEPES pH 7.0, 150 mM KCl, 10% (*v*/*v*) ethylene glycol and various concentrations of each compound was prepared in 96-well PCR plates (ThermoFisher Scientific, Waltham, MA, USA) on ice. The plates were centrifuged to remove air bubbles and then measured by using the Applied Biosystems real-time PCR system (ThermoFisher Scientific, Waltham, MA, USA) with an excitation filter of 580 ± 10 nm and an emission filter of 623 ± 14 nm. The plates were gradually heated from 4 °C to 95 °C at a rate of 0.05 °C/s. The melting curves were fitted with a Boltzmann model using the Protein Thermal Shift software to calculate the protein melting temperature (T_m_). Due to the compounds containing a cysteine group, which is easily oxidized, forming a disulfide, the reducing agent TCEP (1 mM) was added to eliminate the effects of oxidation. The compared results are shown in Table 1. Triplicate assays were applied to controls, and all compounds and the averaged T_m_ were used.

### 3.7. Antibacterial Activity Measurements

*Mycobacterium tuberculosis* H37Ra ATCC 25177 (*Mtb*), a *Mtb Δmtr::Hyg* derivative strain and *Mycobacterium abscessus* ATCC 19977 (*Mab*) containing an episomal plasmid with PSMT-1 were routinely cultured in 7H9 supplemented with 10% (*v/v*) albumin-dextrose-saline (ADS), 0.2% (*v/v*) glycerol and 0.5% (*v/v*) tyloxapol at 37 °C. First, the test compounds were dissolved in 100% DMSO to reach a final concentration of 20 mM DMSO. After confirmation of the compounds’ solubility, the test compounds were spotted in a 96-well plate in one-over-three dilution series by complementing with 7H9 supplemented with 10% (*v/v*) ADS, 0.2% (*v/v*) glycerol and 0.5% (*v/v*) tyloxapol to reach the final concentration starting from 100 µM with a maximum of 1% (*v/v*) DMSO. As a positive control, moxifloxacin was added. Solubility in aqueous conditions was confirmed visually. Next, the plates containing the *Mtb* strains were inoculated at an OD_600_ of 0.05, and the *Mab* strain was inoculated at an RLU of 1 × 10^4^; they were subsequently incubated for 7 days (*Mtb*) or 3 days (*Mab*) at 37 °C. The viability of both *Mtb* and *Mab* was measured as a reduction in luminescence compared to the untreated reference culture by using a Promega discover multi-well plate reader (Promega, Madison, WI, USA). For the *Mtb* strains, the BacTiter-GloTM Microbial Cell Viability Assay (Promega, Madison, WI, USA) was used to provide a luminescent signal corresponding to the amount of ATP present.

## 4. Conclusions

Aminoacyl-sulfonamide-based compounds have been reported for class I LeuRS and class II ThrRS possessing potent antibacterial activity and species selectivity. Since MshC shares a similar catalytic pocket to class I CysRS, it stimulated us to generate similar compounds targeting the former protein, being essential in the regulation of oxidative stress in *Mycobacterium*. Based on this rational design, we reported a new series of cysteinyl-sulfonamide-based compounds that can effectively target *Mycobacterium* MshC. Two positive hits (compound **7b** and **7d**) were identified and suggested that heterocyclic substitutions at the meta position of the phenyl moiety are favorable for binding with MshC compared with a phenyl moiety. Additionally, the full-length crystal structures of ligand-free and compound-bound MshC provide the first glimpse of how this sulfonamide scaffold induces a number of conformational changes in the protein upon binding in the catalytic site. This result likely also reflects the binding mode of *N*-leucyl sulfonamide inhibitors targeting class I LeuRS [25]. Although the anti-mycobacterium activities of these compounds still need to be improved, the general synthetic route described can provide the basis for the production of a broad range of meta-substituted MshC inhibitors based on this cysteinyl phenylsulfonamide scaffold. In parallel, the current ligand-bound MshC structure provides good visual insights for further optimization.

## Figures and Tables

**Table 1:** Thermal stabilization capability of the synthesized compounds against Ms-MshC.

Compounds	Conc. (µM)	T_m_ (°C) ^1^	∆T_m_ (°C)	T_m_ (°C; + 1mM TCEP) ^5^	∆T_m_ (°C) ^6^	EC_50_
Control	/	58.47 ± 0.07	/	/	/	/
	DMSO 10% (*v/v*)	55.09 ± 0.98	−3.38 ^2^	/	/	/
	EDO 10% (*v/v*)	57.99 ± 0.04	−0.48 ^3^	57.91 ± 0.05	/	/
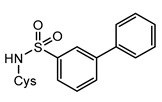 **7a**	1000	57.56 ± 0.16	−0.43 ^4^	57.46 ± 0.26	−0.45	/
	100	58.05 ± 0.12	+0.06 ^4^	58.00 ± 0.09	+0.09	
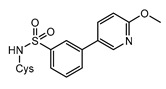 **7b**	1000	58.08 ± 0.23	+0.09 ^4^	60.99 ± 0.23	+3.08	219.29 ± 1.27
	100	57.98 ± 0.08	−0.01 ^4^	59.15 ± 0.05	+1.24	
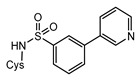 **7c**	1000	58.20 ± 0.14	+0.21 ^4^	58.50 ± 0.15	+0.59	/
	100	58.01 ± 0.07	+0.02 ^4^	57.90 ± 0.01	−0.01	
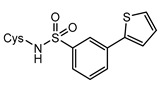 **7d**	1000	58.98 ± 0.22	+0.99 ^4^	61.66 ± 0.29	+3.75	230.60 ± 2.16
	100	57.94 ± 0.09	−0.05 ^4^	59.01 ± 0.22	+1.10	
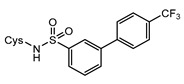 **7e**	1000	58.13 ± 0.14	+0.14 ^4^	58.67 ± 0.17	+0.76	/
	100	57.76 ± 0.08	−0.23 ^4^	57.76 ± 0.03	−0.15	
L-Cys	10,000	61.56 ± 0.06	+3.09 ^4^	/	/	/
	1000	59.20 ± 0.07	+0.73 ^4^	/	/	
ATP	10,000	59.82 ± 0.18	+1.35 ^4^	/	/	/
	1000	58.20 ± 0.03	−0.27 ^4^	/	/	
Cys-AMP	1000	66.55 ± 0.13	+8.08 ^4^	/	/	106.64 ± 1.34
	100	62.23 ± 0.13	+3.76 ^4^	/	/	

^1^ All thermal shift assays were performed in triplicates. The T_m_ values are shown as average number ± standard deviations for triplicate measurements. ^2^ ∆T_m_ was measured by the difference of Ms-MshC in buffer and in buffer supplemented with 10% (*v*/*v*) DMSO. ^3^ ∆T_m_ was measured by the difference of Ms-MshC in buffer and in buffer supplemented with 10% (*v*/*v*) EDO. ^4^ ∆T_m_ was measured by the difference of Ms-MshC with and without ligand in buffer containing 10% (*v*/*v*) EDO. ^5^ T_m_ values of Ms-MshC were measured without or with ligand in buffer containing 10% (*v*/*v*) EDO and 1 mM TCEP. The presence of TCEP ensures the compounds in the reduced form to eliminate the effect of the compound auto-oxidation. ^6^ ∆T_m_ was measured by the difference of Ms-MshC with and without ligand in buffer containing 10% (*v*/*v*) EDO and 1mM TCEP.

**Table 2:** X-ray diffraction data collection and refinement statistics of Ms-MshC and Ms-MshC-ligand complexes.

	MshC (apo)	MshC-Compound 7b	MshC-Compound 7d
PDB Code	8HFM	8HFN	8HFO
Data collection			
Resolution range (Å)	48.32–2.41 (2.50–2.41)	43.68–1.98 (2.05–1.98)	55.03–2.773 (2.87–2.77)
Space group	P 4_1_ 2 2	P 6_1_	P 6_1_
Unit cell			
a, b, c (Å)	69.81 69.81 236.01	166.02 166.02 51.36	168.11 168.11 52.30
α, β, γ (°)	90 90 90	90 90 120	90 90 120
Unique reflections	23,525 (2275)	56,710 (5394)	21,800 (2176)
Multiplicity	26.2 (26.6)	10.2 (10.6)	20.1 (20.7)
Completeness (%)	99.9 (100.0)	99.9 (99.9)	99.9 (99.9)
Mean *I*/σ (*I*)	15.3 (1.6)	14.1 (2.6)	19.0 (2.3)
Wilson B-factor (Å^2^)	56.4	41.2	92.4
*R*_merge_	0.167 (1.995)	0.086 (1.160)	0.098 (1.438)
*R*_meas_	0.171 (2.034)	0.090 (1.219)	0.100 (1.474)
*R*_pim_	0.033 (0.393)	0.029 (0.373)	0.023 (0.323)
CC_1/2_	0.998 (0.760)	0.998 (0.886)	0.993 (0.889)
Refinement			
Reflections used for refinement	23,514 (2276)	53,801 (5391)	21,784 (2174)
*R*_work_	0.207 (0.298)	0.199 (0.490)	0.213 (0.409)
*R*_free_	0.238 (0.359)	0.223 (0.460)	0.258 (0.356)
Number of non-H atoms	3197	3473	3214
Macromolecules	3130	3200	3181
Inhibitor	/	27	23
Solvent	65	246	10
RMS bonds (Å)	0.004	0.004	0.005
RMS angles (°)	0.66	0.77	0.89
Ramachandran favoured (%)	98.25	99.27	95.38
Ramachandran allowed (%)	1.75	0.73	4.38
Average B-factor (Å^2^)	65.9	53.64	109.07
Protein	66.06	53.55	109.27
Inhibitor	/	46.85	82.91
Solvent	57.98	55.63	105.87

Statistics were generated using Phenix [28]; values in parenthesis correspond to the highest resolution shell.

**Table 3:** Activity of the test compounds (**7a**–**e**) against *Mycobacterium tuberculosis* and *Mycobacterium abscessus*.

Compound	pIC_50_ ^a^		
	*Mtb* ^b^		*Mab* ^c^
	WT ^d^	*Δmtr*::Hyg ^e^	WT
**7a**	<3.9	<3.9	<3.9
**7b**	<3.9	<3.9	<3.9
**7c**	<3.9	<3.9	<3.9
**7d**	<3.9	<3.9	<3.9
**7e**	<3.9	<3.9	<3.9
^f^ MXF	8.1	6.3	5.5

^a^ pIC_50_: calculated as the negative log of the IC_50_ value when converted to molar. ^b^ *Mtb*: *Mycobacterium tuberculosis* H37Ra ATCC^TM^ 25177. ^c^ *Mab*: *Mycobacterium abscessus* ATCC^TM^ 19977 containing an episomal plasmid with PSMT-1. ^d^ WT: wild type. ^e^ *Δmtr::Hyg*, *Mtb* H37Ra derivative strain carrying a mycothiol reductase gene replaced with a hygromycin resistance cassette. ^f^ MXF: moxifloxacin, a second line of care injectable antibiotic with activity against *Mtb* and *Mab* used as a positive control.

## Data Availability

All structural datasets generated in this work are available in the PDB repository (https://www.rcsb.org/ (accessed on 10 November 2022)) under accession codes 8HFM, 8HFN and 8HFO.

## Supplementary Materials

The following supporting information can be downloaded at: https://www.mdpi.com/article/10.3390/ijms232315095/s1.
