# Supplementary material for: Structural Basis of Cysteine Ligase MshC Inhibition by Cysteinyl-Sulfonamides
Source: Int J Mol Sci. 2022 Dec 1;23(23):15095. doi: 10.3390/ijms232315095 (PMC9736012; doi:10.3390/ijms232315095)
Supplement: Supplementary file 1 [file ijms-23-15095-s001.zip › ijms-2058493-supplementary.pdf]

Supplementary file

Synthetic procedures and chemical analysis of all compounds

1. General procedures

General procedure A

3-bromobenzenesulfonamide (300 mg, 1.26 mmol, 1 eq), the respective boronic acid (1.4 mmol, 1.1 eq) and K_2CO_3 (538 mg, 3.9 mmol, 3.1 eq) are dissolved in 15 mL of a 4:1 mixture of 1,4-dioxane:water. Pd(dppf)$(Cl)_2$ (175 mg, 0.25 mmol, 0.2 eq) is added and the reaction mixture is stirred overnight at 110 °C. After consumption of the starting material, the reaction is cooled down to room temperature and diluted with 30 mL of MeOH. The mixture is filtered over a celite plug and dried using anhydrous Na_2SO_4. The mixture is filtered again, and the solvents are evaporated to dryness. The resulting crude is purified using silica gel chromatography.

General procedure B

The sulfonamide (1 eq), N-(tert-butoxycarbonyl)-S-tritylcysteine (1.2 eq), O-Benzotriazole-N,N,N',N'-tetramethyluronium-hexafluoro-phosphate (HBTU, 1.5 eq) and triethylamine (3 eq) are dissolved in 4 mL of DMF and stirred overnight. After TLC showed full comsumption of the starting material, the reaction mixture is diluted with 25 mL of ethyl acetate (EtOAc) and washed with 3x 25 mL of brine. The organic phase is dried using anhydrous Na_2SO_4, filtered and evaporated to dryness. The resulting crude is purified using silica gel chromatography.

General procedure C

An aliquot of 1 mL of trifluoroacetic acid (TFA) is added to a solution of the starting material in 4 mL of dichloromethane (DCM). Triethylsilane (2.5 eq) is added, and the solution stirred for 1 h. The reaction is quenched with saturated $NaHCO_3$ and extracted using DCM. The organic phases are collected, dried using anhydrous Na_2SO_4 and filtered. The organic phase is evaporated to dryness, and the resulting crude purified using silica gel chromatography.

2. Synthesis and analysis of obtained compounds

2.1. [1,1'-biphenyl]-3-sulfonamide (5a)

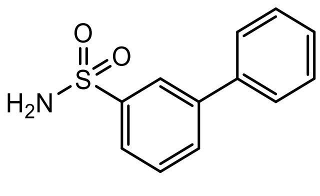

General procedure A is followed using phenylboronic acid (170 mg). The crude is purified using silica gel chromatography (30% of ethyl acetate (EtOAc) in heptane) to obtain the title compound as an off-white powder (240 mg, 82% yield).

^{1}H NMR (300 MHz, $CDCl_3$): δ (ppm) = 8.16 (s, 1H), 7.91 (d, J= 8.75Hz, 1H), 7.81 (d, J= 8.75 Hz, 1H) 7.65-7.56 (m, 3H), 7.52-7.37 (m, 3H), 4.82 (s, 2H, NH); ^{13}C NMR (150 MHz, $(CD_3)_2SO$): δ (ppm) = 144.88, 140.90, 138.83, 129.99, 129.72, 129.24,128.07, 126.83, 124.44, 123.81.

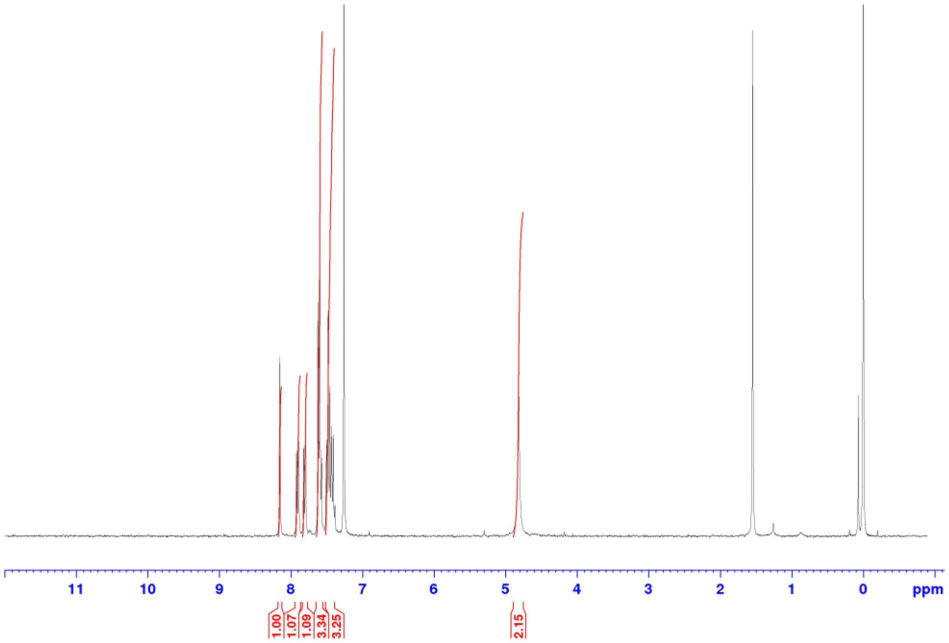

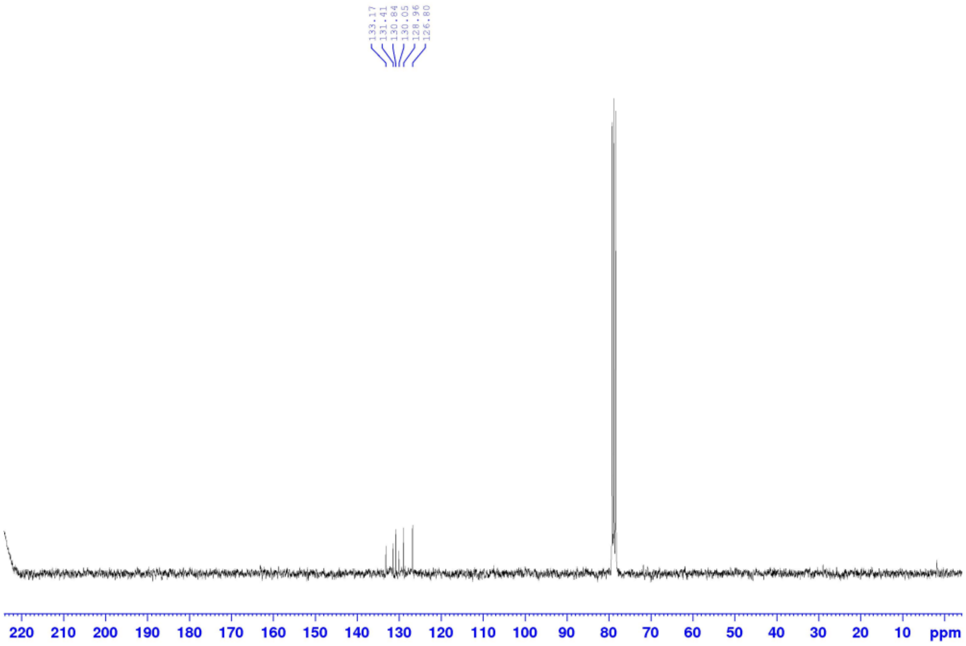

2.2. 3-(6-methoxypyridin-3-yl)benzenesulfonamide (5b)

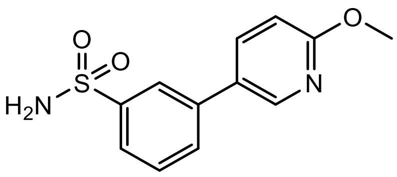

General procedure A is followed using (6-methoxypyridin-3-yl)boronic acid (214 mg). The crude is purified using silica gel chromatography (40% EtOAc in heptane) to obtain the title compound as an off-white powder (248 mg, 74% yield).

^{1}H NMR (300 MHz, $CDCl_3$): δ (ppm) = 8.41 (d, J= 3.3 hz, 1H), 8.08 (s, 1H), 7.90 (d, J= 8.01 Hz, 1H), 7.80 (dd, J= 2.5 Hz, 8.01 Hz, 1H), 7.74 (d, 8.01 Hz, 1H), 7.60 (t, J= 8.01 Hz, 1H), 6.85 (d, J= 8.89 Hz), 4.88 (s, 2H, NH), 3.99 (s, 3H); ^{13}C NMR (75 MHz, $CDCl_3$): δ (ppm) = 145.24, 137.36, 130.83, 129.89, 128.23, 125.07, 124.52, 111.20, 53.71; HRMS (ESI): calcd. for $C_{12}H_{13}N_2O_3S$ $[M+H]^+$: 265.0641, found: 265.0641.

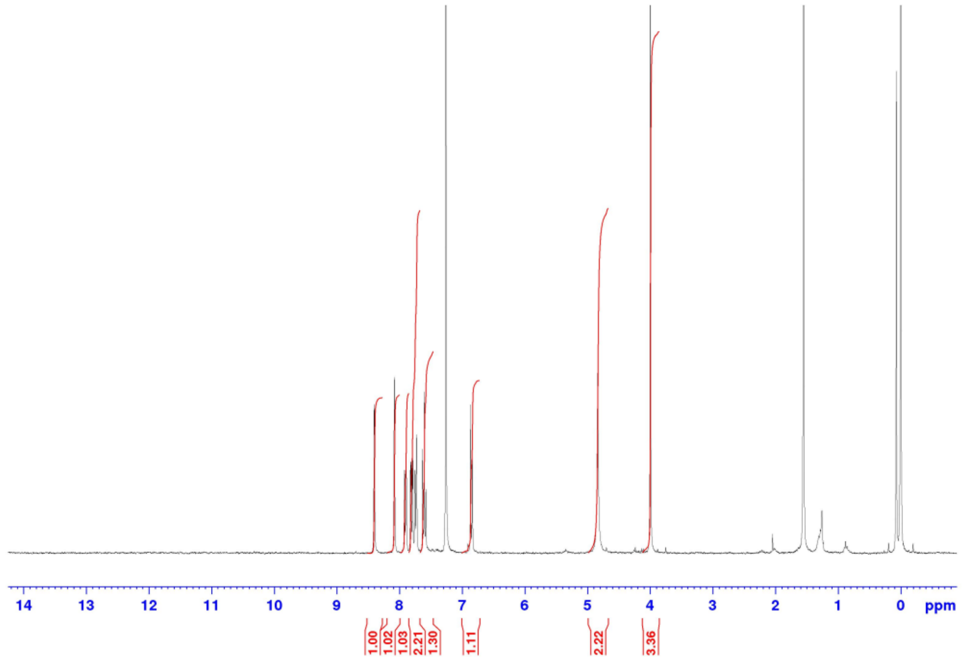

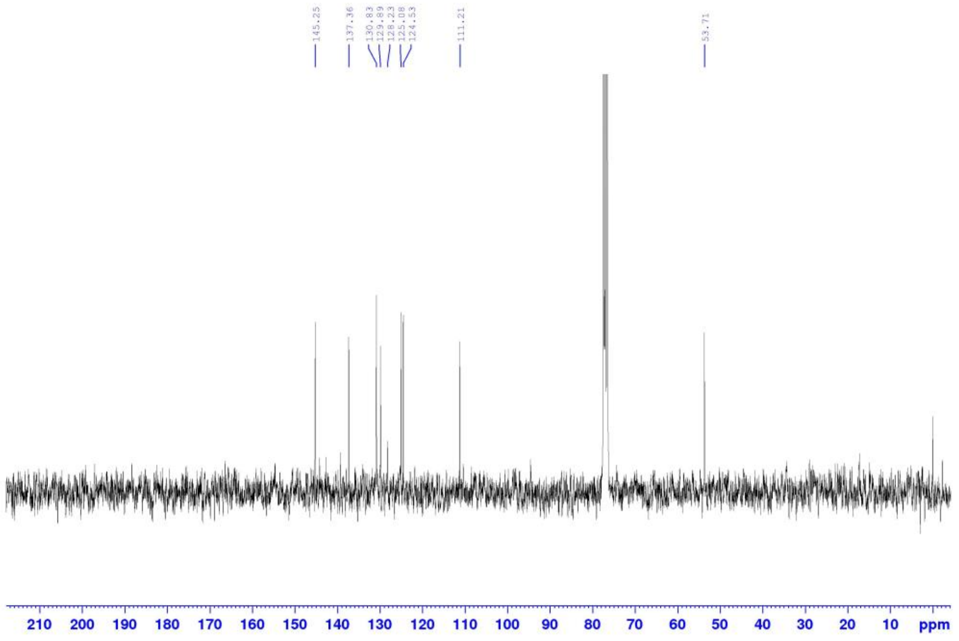

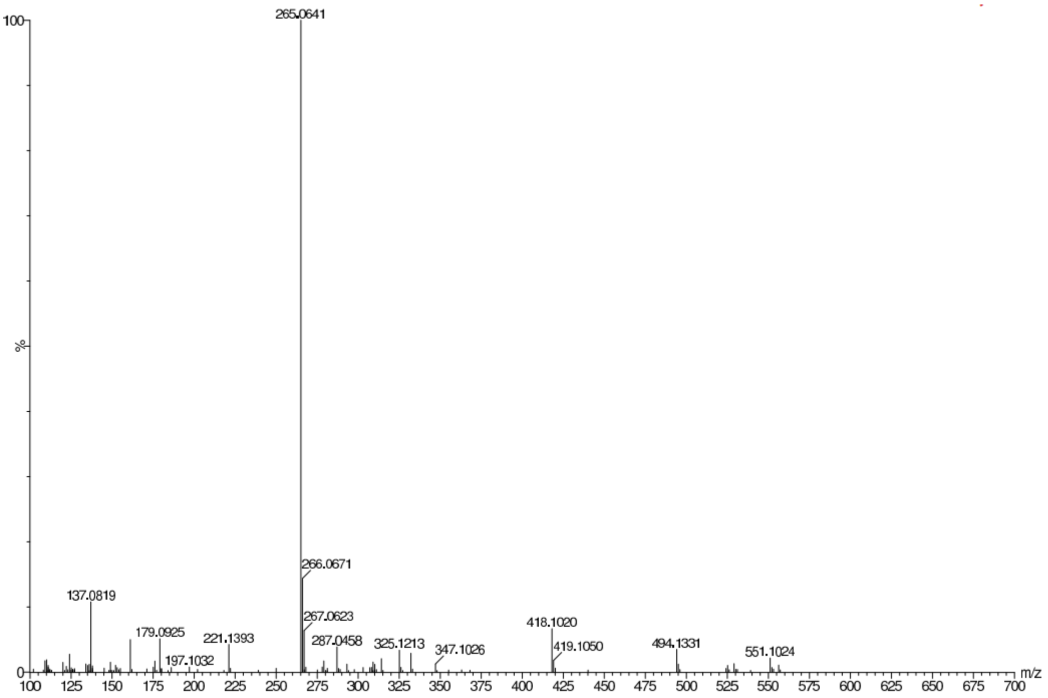

2.3. 3-(pyridin-3-yl)benzenesulfonamide (5c)

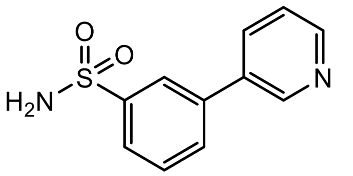

General procedure A is followed using pyridin-3-ylboronic acid (172 mg). The crude is purified using silica gel chromatography (60% EtOAc in heptane) to obtain the title compound as a brown powder (192 mg, 65% yield).

^{1}H NMR (300 MHz, MeOD): δ (ppm) = 8.86 (d, J= 2.22 Hz, 1H), 8.58 (dd, J= 1.40 Hz, 4.45 Hz, 1H), 8.22-8.13 (m, 2H), 7.96 (d, J=7.49 Hz, 1H), 7.91 (d, J=7.49 Hz, 1H), 7.69 (t, J= 8.02Hz, 1H), 7.60-7.53 (m, 1H); ^{13}C NMR (75 MHz, MeOD): δ (ppm) = 131.78, 129.18, 128.77, 127.90, 127.57, 126.83, 126.45, 126.07; HRMS (ESI): calcd. for $C_{11}H_{11}N_2O_2S$ $[M+H]^+$: 235.0535, found: 235.0534.

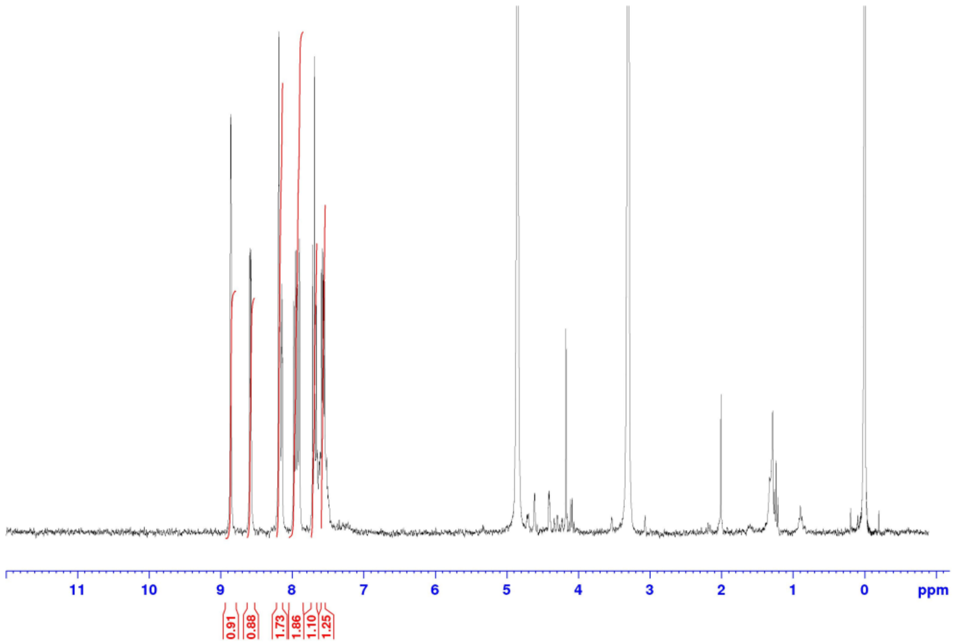

0.91
0.88
1.73
1.86
1.10
1.25
11 10 9 8 7 6 5 4 3 2 1 0 ppm

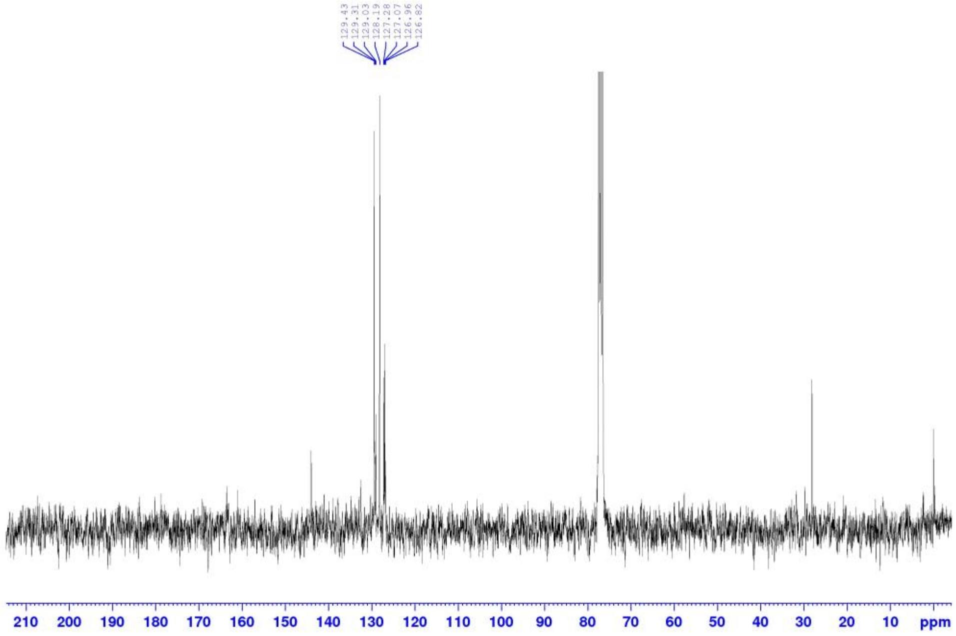

129.43
129.31
129.03
128.19
127.28
127.07
126.96
126.82
210 200 190 180 170 160 150 140 130 120 110 100 90 80 70 60 50 40 30 20 10 ppm

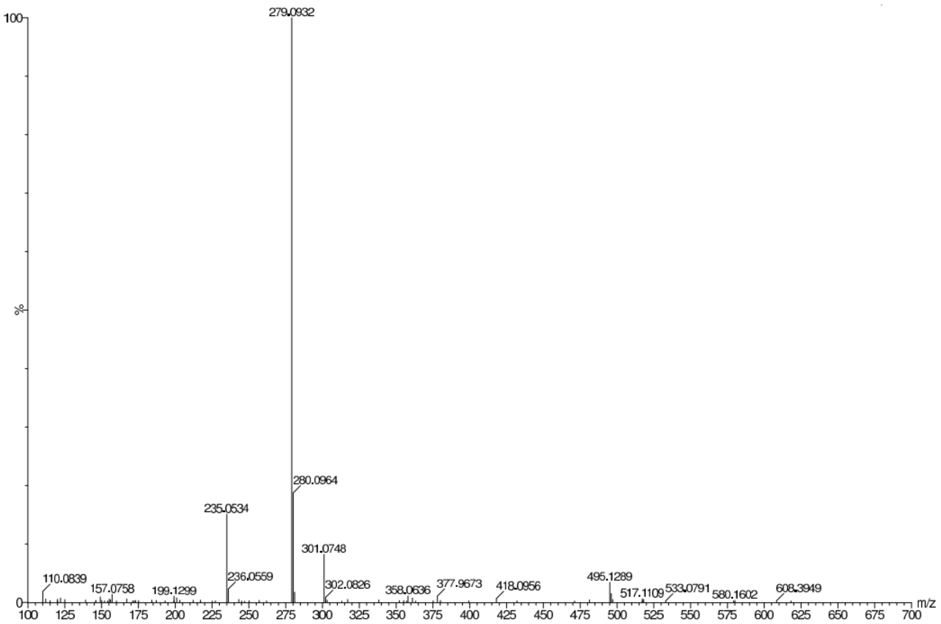

2.4. 3-(thiophen-2-yl)benzenesulfonamide (5d)

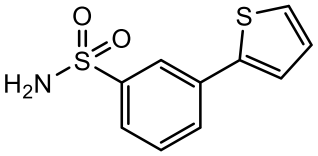

General procedure A is followed using thiophen-2-ylboronic acid (179 mg). The crude is purified using silica gel chromatography (50% EtOAc in heptane) to obtain the title compound as a brown powder (214 mg, 71% yield).

^{1}H NMR (300 MHz, MeOD): δ (ppm) = 8.17 (s, 1H), 7.87 (d, J= 8.26Hz, 1H), 7.81 (d, J= 8.26Hz, 1H), 7.75-7.71 (m, 1H), 7.59-7.47 (m, 3H); ^{13}C NMR (75 MHz, MeOD): δ (ppm) = 144.25, 140.49, 136.7, 129.40, 129.22, 126.58, 125.55, 124.01, 123.27, 121.32.

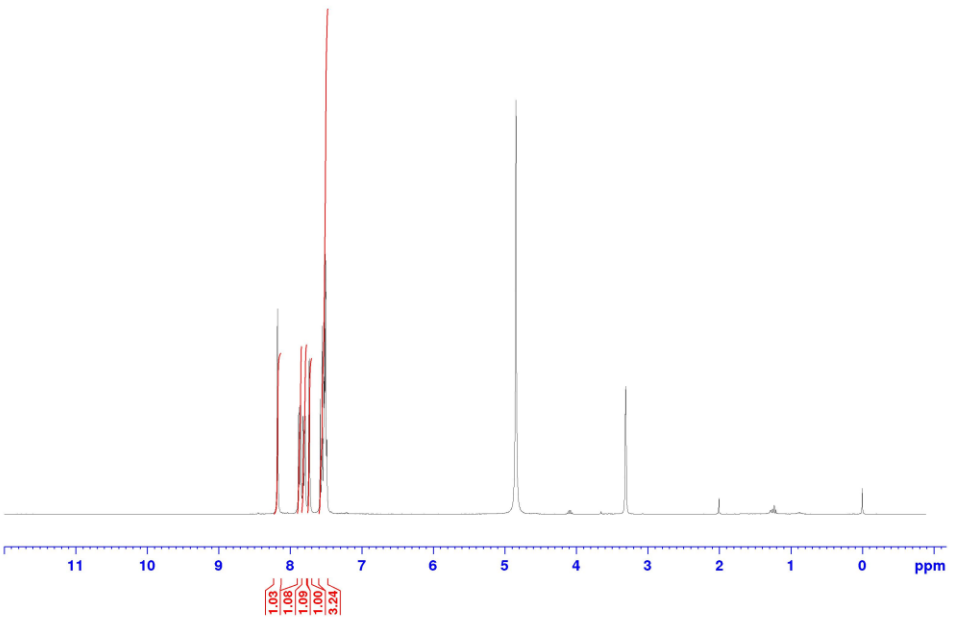

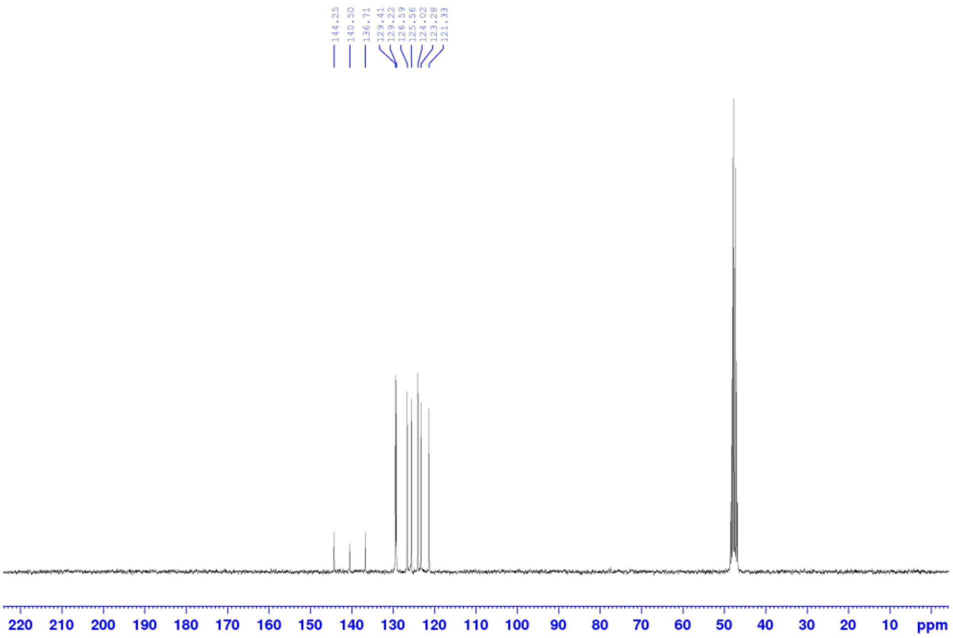

2.5. 4'-(trifluoromethyl)-[1,1'-biphenyl]-3-sulfonamide (5e)

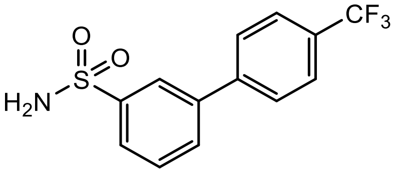

General procedure A is followed using (4-(trifluoromethyl)phenyl)boronic acid (266 mg). The crude is purified using silica gel chromatography (30% EtOAc in heptane) to obtain the title compound as a white powder (251 mg, 66% yield).

^{1}H NMR (300 MHz, (MeOD): δ (ppm) = 8.20 (s, 1H), 7.98-7.84 (m, 4H),7.82-7.75 (m, 2H), 7.67 (t, J= 7.80, 1H); ^{13}C NMR (150 MHz, (MeOD): δ (ppm) = 144.64, 143.28, 140.36, 130.41, 129.48, 127.38, 125.65, 125.60, 125.37, 124.37; MS (ESI): calcd. for $C_{13}H_{11}F_{3}NO_{2}S$ [M+H]$^{+}$: 302.0, found: 301.9.

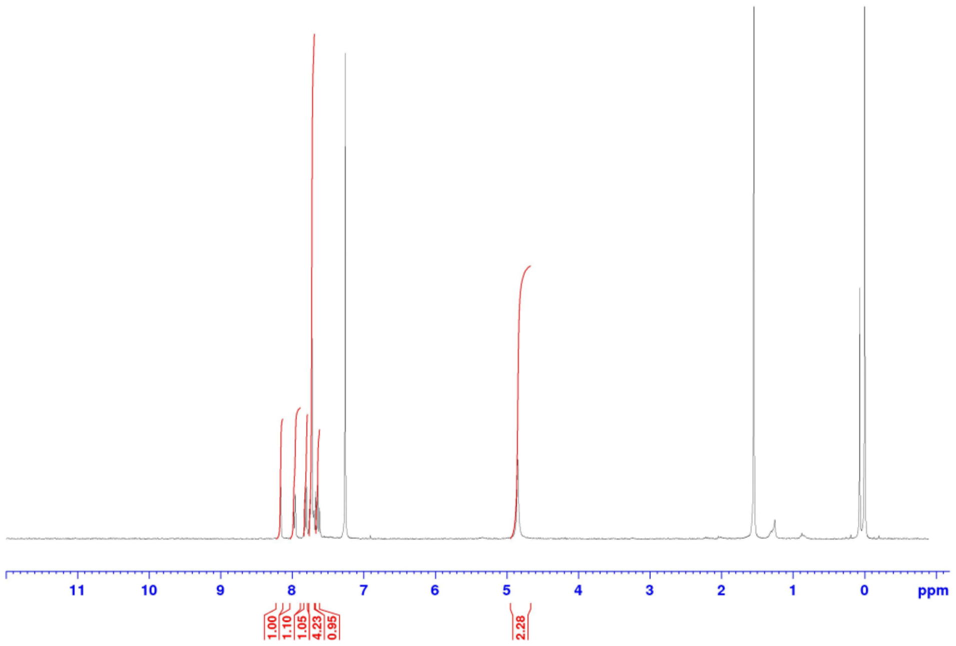

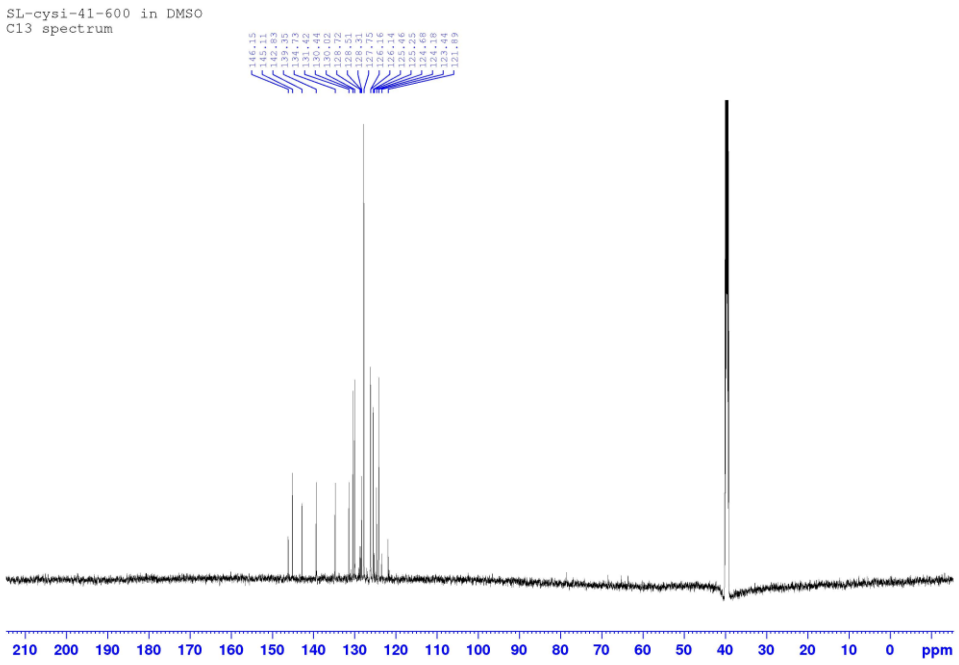

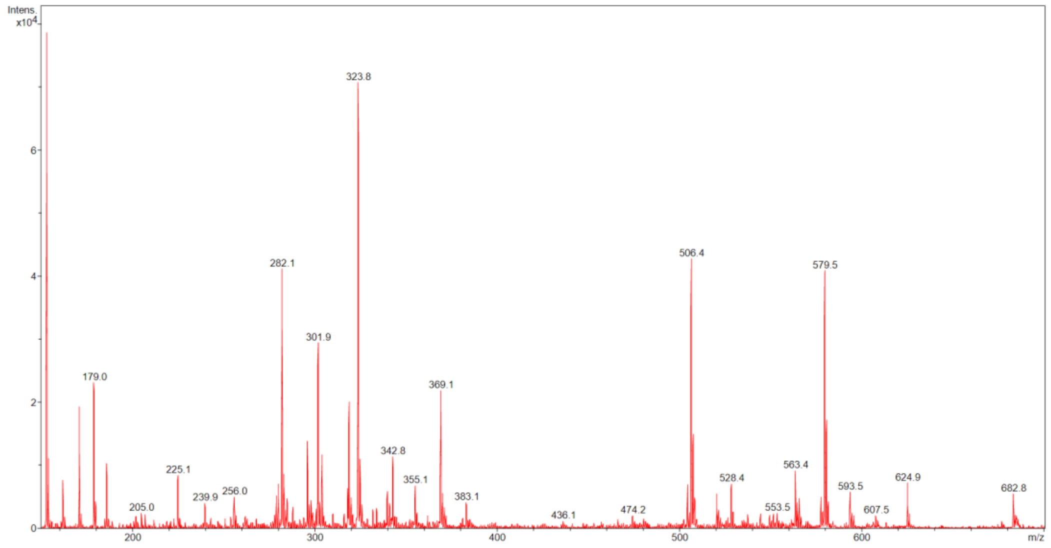

2.6. tert-butyl (1-([1,1'-biphenyl]-3-sulfonamido)-1-oxo-3 (R) -(tritylthio)propan-2-yl)carbamate (6a)

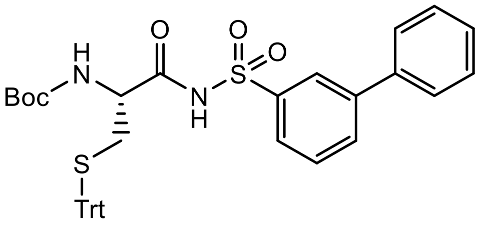

General procedure B was followed using **5a** [1,1'-biphenyl]-3-sulfonamide (240 mg, 1.03 mmol), N-(tert-butoxycarbonyl)-S-tritylcysteine (574 mg, 1.24 mmol), HBTU (590 mg, 1.55 mmol) and triethylamine (458 µL, 3.09 mmol). The crude is purified using silica gel chromatography (30% EtOAc in heptane) to obtain the title compound **6a** as a yellow powder (149 mg, 21% yield).

^{1}H NMR (300 MHz, (MeOD): δ (ppm) = 8.23 (s, 1H), 7.94 (d, J= 8.23 Hz, 1H), 7.84 (d, J= 7.90 Hz, 1H), 7.64 ‘s, 1H), 7.61 (s, 1H), 7.55 (t, J= 8.23 Hz), 7.49-7.35 (m, 3H), 7.26-7.15 (m, 15H), 2.30 (d, J= 6.53 Hz, 2H), 1.35 (s, 9H), 0.98 (t, J= 6.53 Hz, 1H); ^{13}C NMR (75 MHz, (MeOD): δ (ppm) = 144.23, 131.75, 129.18, 128.77, 127.90, 127.57, 126.83, 126.45, 126.07, 27.16 HRMS (ESI): calcd. for $C_{39}H_{38}N_2O_5S_2Na$ [M+Na]$^+$: 701.2114, found: 701.2131; calcd. for $C_{39}H_{37}N_2O_5S_2$ [M-H]$^-$: 677.21492, found: 677.2141.

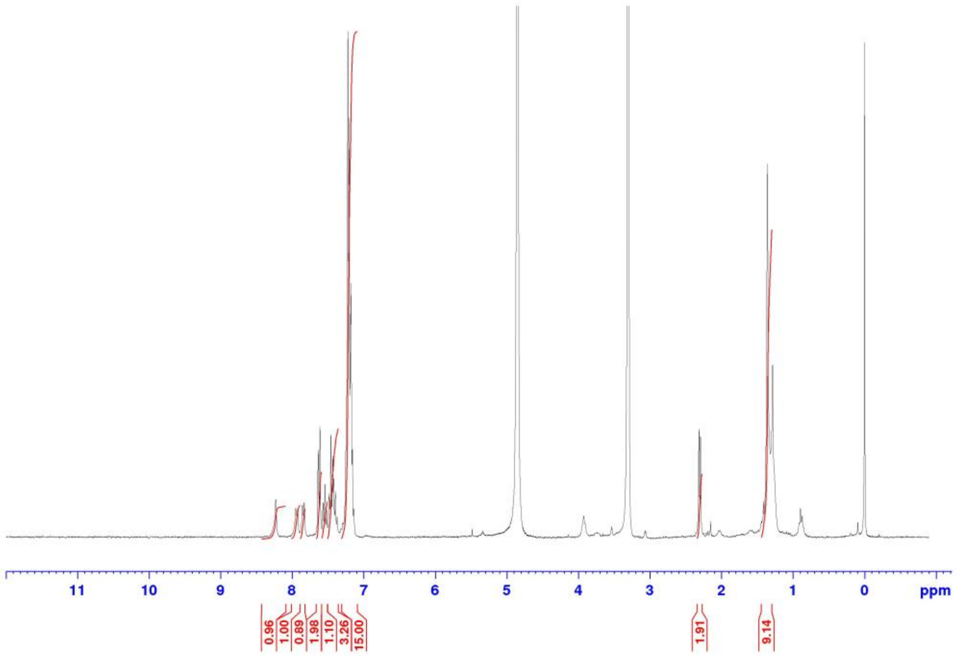

11
10
9
8
7
6
5
4
3
2
1
0
ppm
0.96
1.00
0.89
1.98
1.10
3.26
15.00
1.91
9.14

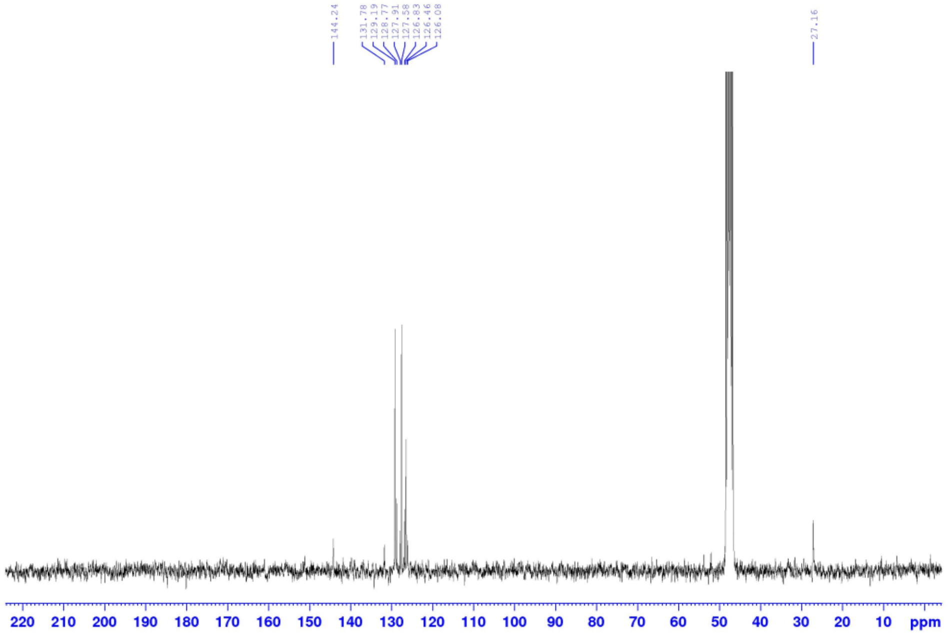

144.24
131.78
129.19
128.77
127.91
127.58
126.83
126.46
126.08
27.16
220
210
200
190
180
170
160
150
140
130
120
110
100
90
80
70
60
50
40
30
20
10
ppm

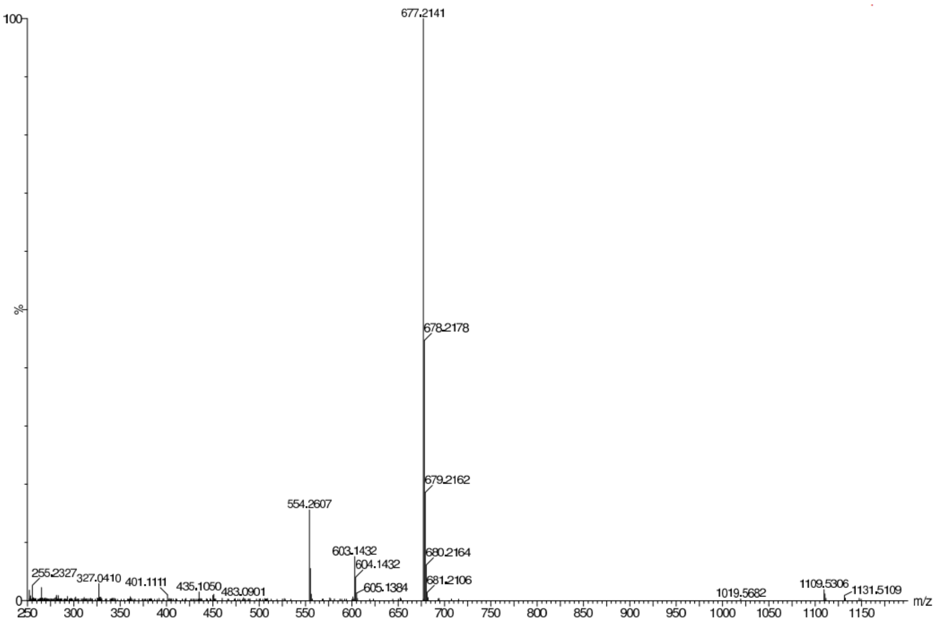

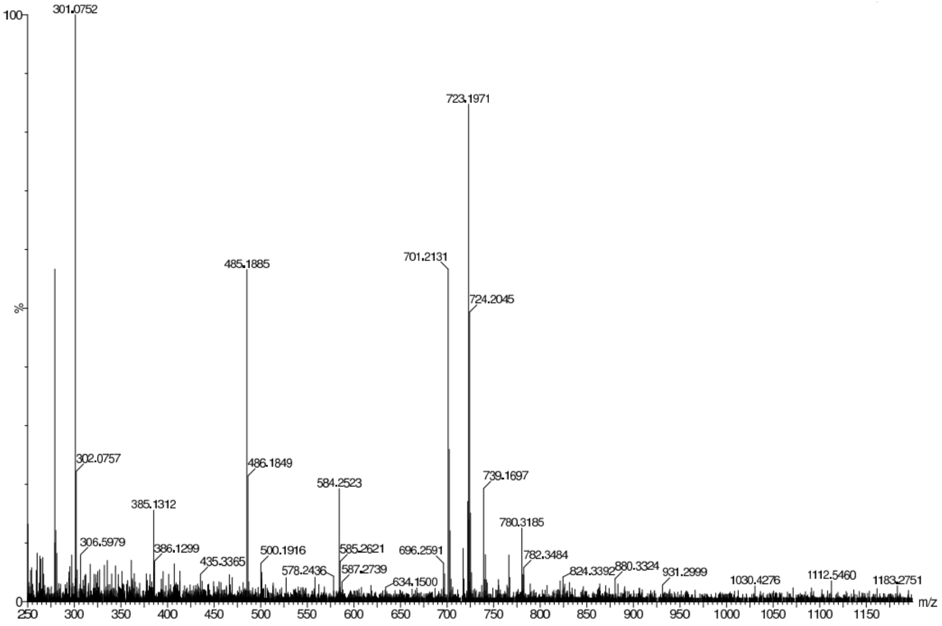

2.7. tert-butyl (1-((3-(6-methoxypyridin-3-yl)phenyl)sulfonamido)-1-oxo-3 (R)-(tritylthio)propan-2-yl)carbamate (6b)

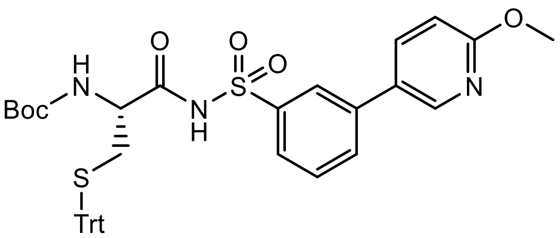

General procedure B was followed using **5b** (248 mg, 0.93 mmol), N-(tert-butoxycarbonyl)-S-tritylcysteine (534 mg, 1.12 mmol), HBTU (531 mg, 1.40 mmol) and triethylamine (412 µL, 2.78 mmol). The crude is purified using silica gel chromatography (30% EtOAc in heptane) to obtain the title compound **6b** as a yellow powder (163 mg, 23% yield).

^{1}H NMR (300 MHz, (MeOD): δ (ppm) = 8.37 (d, J= 2.42 Hz, 1H), 8.20 (s, 1H), 8.98-7.87 (m, 2H), 7.80 (d, J= 8.08 Hz, 1H), 7.76 (t, J= 7.68 Hz, 1H), 7.23-7.16 (m, 15H), 6.86 (d, J= 8.49 Hz, 1H), 3.95 (s, 3H), 2.31 (d, J= 6.76 Hz), 1.35 (s, 9H), 0.87 (t, J= 6.76 Hz, 1H); ^{13}C NMR (75 MHz, (MeOD): δ (ppm) = 170.48, 16.24, 144.81, 144.22, 140.22, 138.45, 137.66, 131.29, 129.17,127.58, 126.48, 125.55, 11.62, 79.65, 66.41, 52.90, 33.18, 27.18, 13.03; HRMS (ESI): calcd. for $C_{39}H_{39}N_3O_6S_2Na$ $[M+Na]^+$: 732.2172, found: 732.2186; calcd. for $C_{39}H_{38}N_3O_6S_2$ $[M-H]^-$: 708.2207, found: 708.2201.

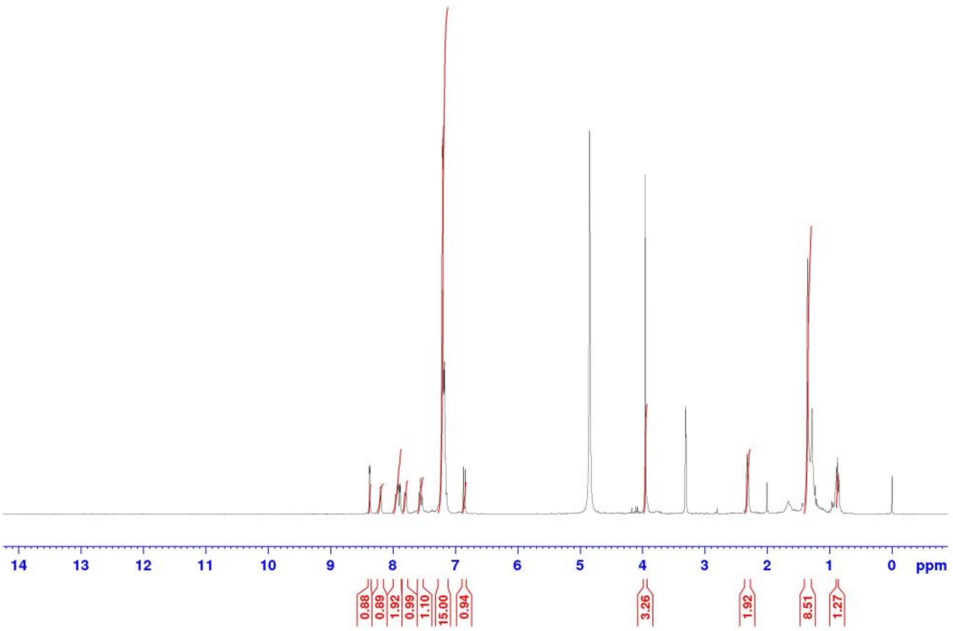

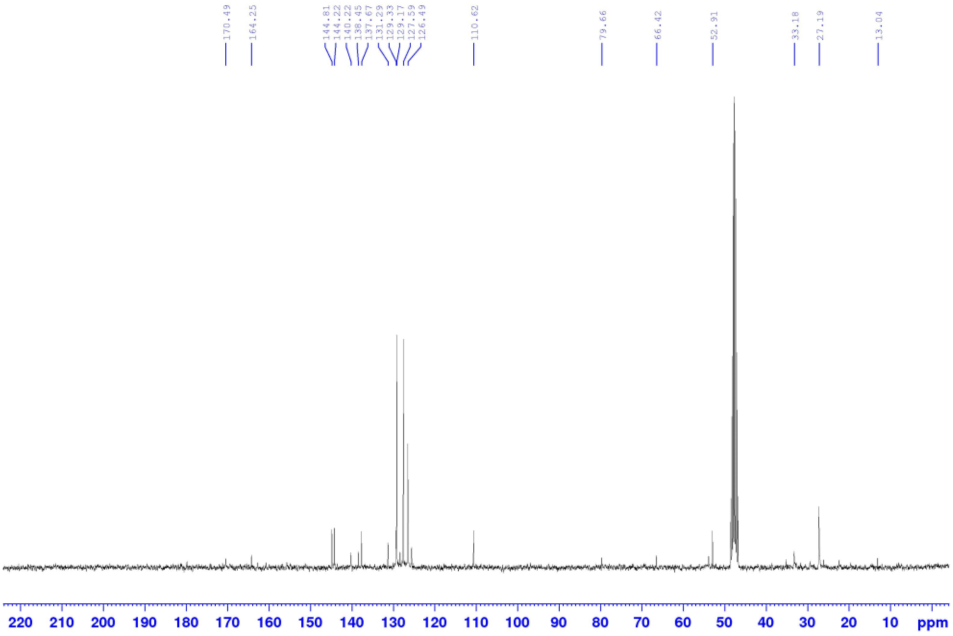

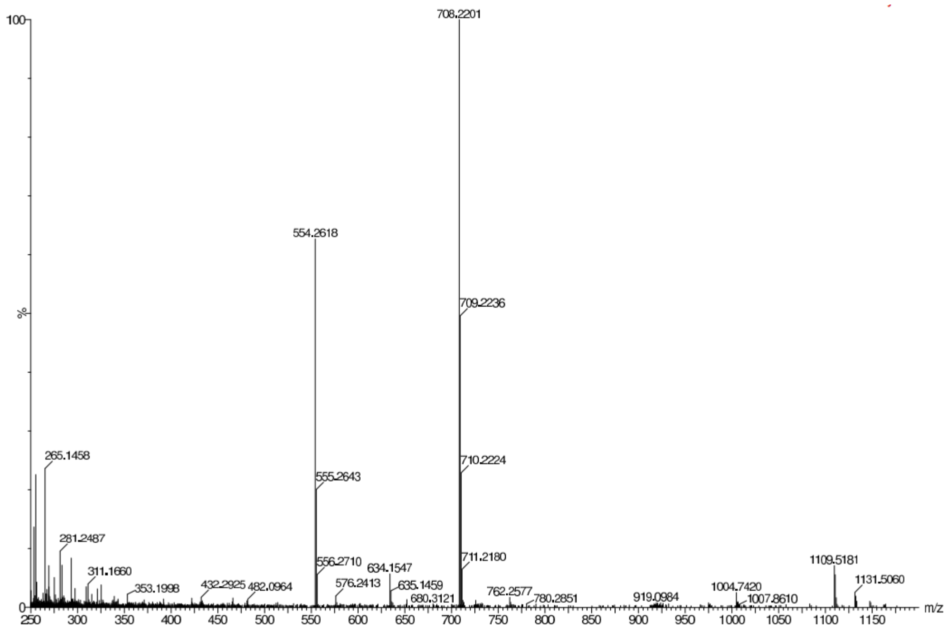

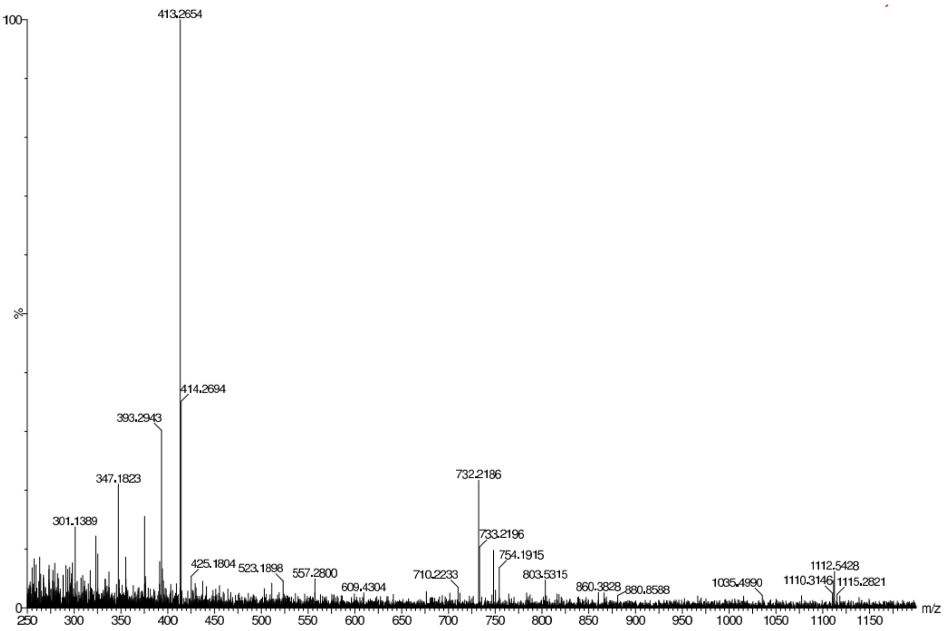

2.8. tert-butyl (1-oxo-1-((3-(pyridin-3-yl)phenyl)sulfonamido)- 3 (R) -(tritylthio)propan-2-yl)carbamate (6c)

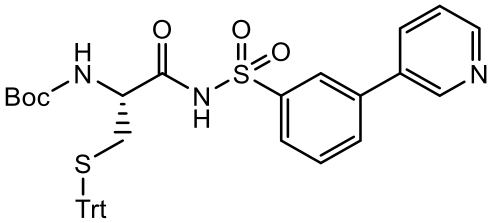

General procedure B was followed using **5c** (192 mg, 0.81 mmol), N-(tert-butoxycarbonyl)-S-tritylcysteine (456 mg, 0.98 mmol), HBTU (466 mg, 1.22 mmol) and triethylamine (362 µL, 2.44

mmol). The crude is purified using silica gel chromatography (50% EtOAc in heptane) to obtain the title compound **6c** as a yellow powder (152 mg, 27% yield).

^{1}H NMR (300 MHz, (DMSO): *δ* (ppm) = 8.80 (s, 1H), 8.56 (d, J= 6.29 Hz, 1H), 8.28 (s, 1H), 8.11-7.98 (m, 2H), 7.87 (d, J= 6.29 Hz, 1H), 7.61 (t, J= 6.86 Hz, 1H), 7.51 (dd, J= 5.07 Hz, 7.88 Hz, 1H), 7.24-7.14 (m, 15H), 3.95 (t, J= 6.09 Hz, 1H), 2.32 (d, J= 6.09 Hz, 2H), 1.35 (s, 9H); ^{13}C NMR (75 MHz, (MeOD): *δ* (ppm) = 170.68, 155.69, 148.14, 147.04, 144.24, 140.59, 137.93, 135.51, 131.79, 129.52, 129.17, 127.58, 126.48, 126.30, 124.30, 79.58, 66.36, 53.89, 33.25, 27.19; MS (ESI): calcd. for $C_{38}H_{38}N_3O_5S_2$ $[M+H]^+$: 680.2, found: 680.1

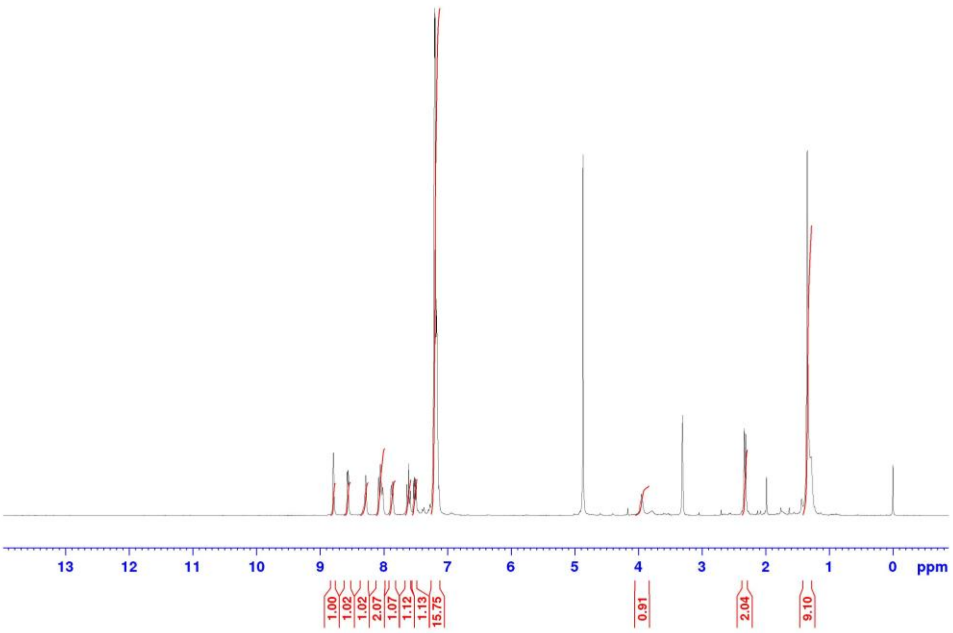

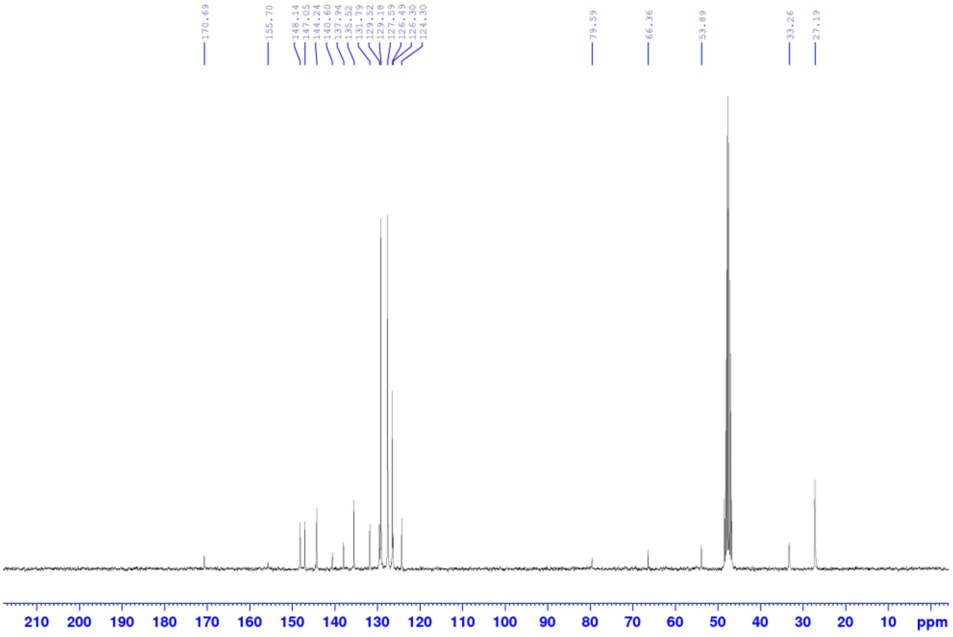

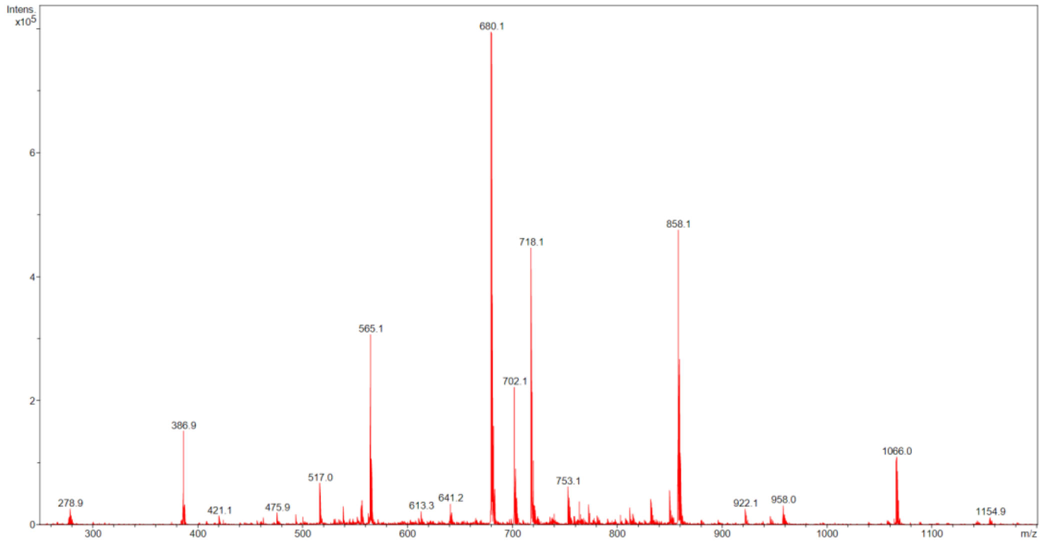

2.9. tert-butyl (1-oxo-1-((3-(thiophen-2-yl)phenyl)sulfonamido)-3 (R) (tritylthio)propan-2-yl)carbamate (6d)

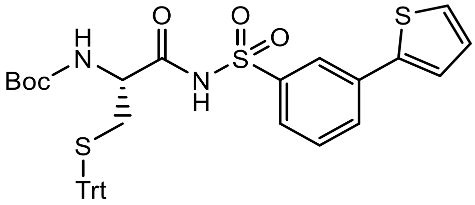

General procedure B was followed using **5d** (214mg, 0.89 mmol), N-(tert-butoxycarbonyl)-S-tritylcysteine (500 mg, 1.08 mmol), HBTU (513 mg, 1.35 mmol) and triethylamine (400 µL, 2.69 mmol). The crude is purified using silica gel chromatography (30% EtOAc in heptane) to obtain the title compound **6d** as a yellow powder (213 mg, 35% yield).

^{1}H NMR (300 MHz, (MeOD): δ (ppm) = 8.26 (s, 1H), 7.86 (d, J= 7.53 hz, 2H), 7.71 (s, 1H), 7.53-7.43 (m, 3H), 7.26-7.13 (m, 15H), 8.92 (t, J= 6.87 Hz, 1H), 2.29 (d, J= 7.24 Hz, 2H), 1.36 (s, 9H); ^{13}C NMR (75 MHz, (MeOD): δ (ppm) = 170.03, 144.19, 140.16, 139.74, 136.65, 131.07, 129.38, 129.18, 127.60, 126.67, 126.49, 126.02, 125.65, 125.26, 121.73, 79.69, 66.47, 53.72, 33.03, 27.18; HRMS (ESI): calcd. for $C_{37}H_{35}N_2O_5S_3$ [M-H]$^-$: 683.1713, found: 683.1708.

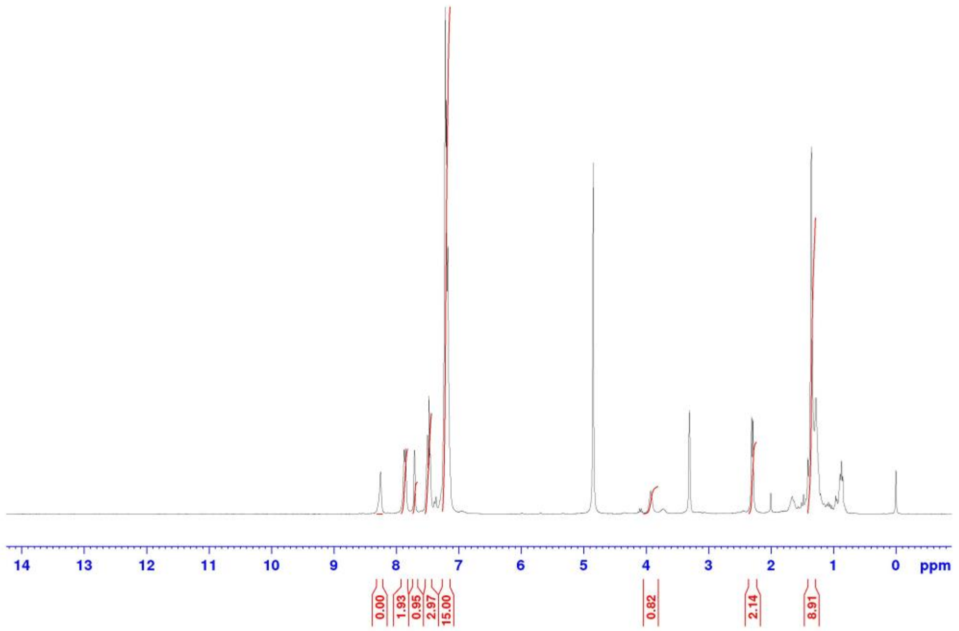

0.00
1.93
0.95
2.97
15.00
0.82
2.14
8.91
14 13 12 11 10 9 8 7 6 5 4 3 2 1 0 ppm

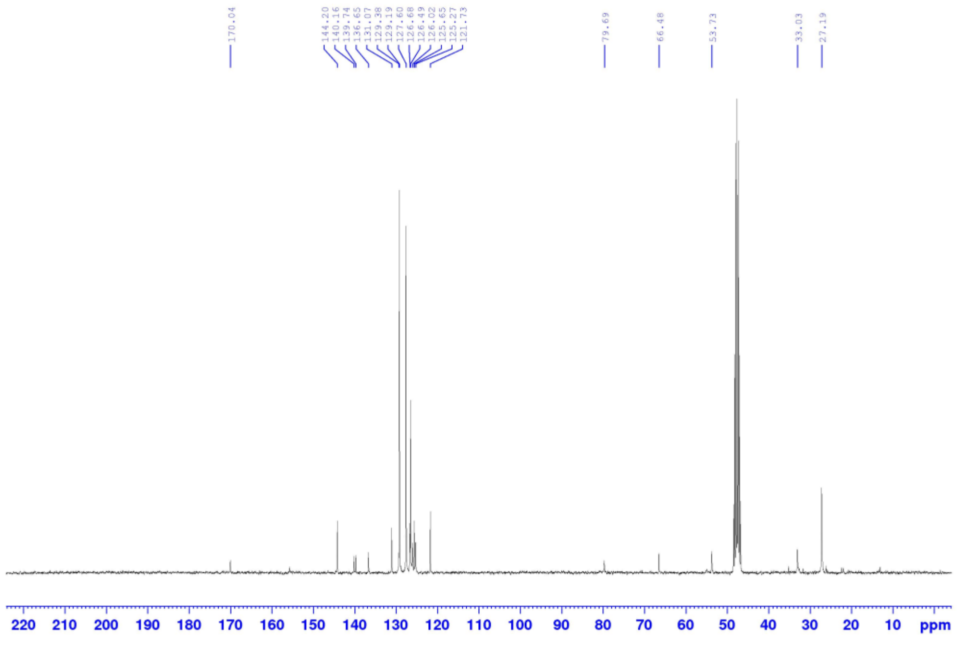

170.04
144.20
140.16
139.74
136.65
131.07
129.38
129.19
127.60
126.68
126.49
126.02
125.65
125.27
121.73
79.69
66.48
53.73
33.03
27.19
220 210 200 190 180 170 160 150 140 130 120 110 100 90 80 70 60 50 40 30 20 10 ppm

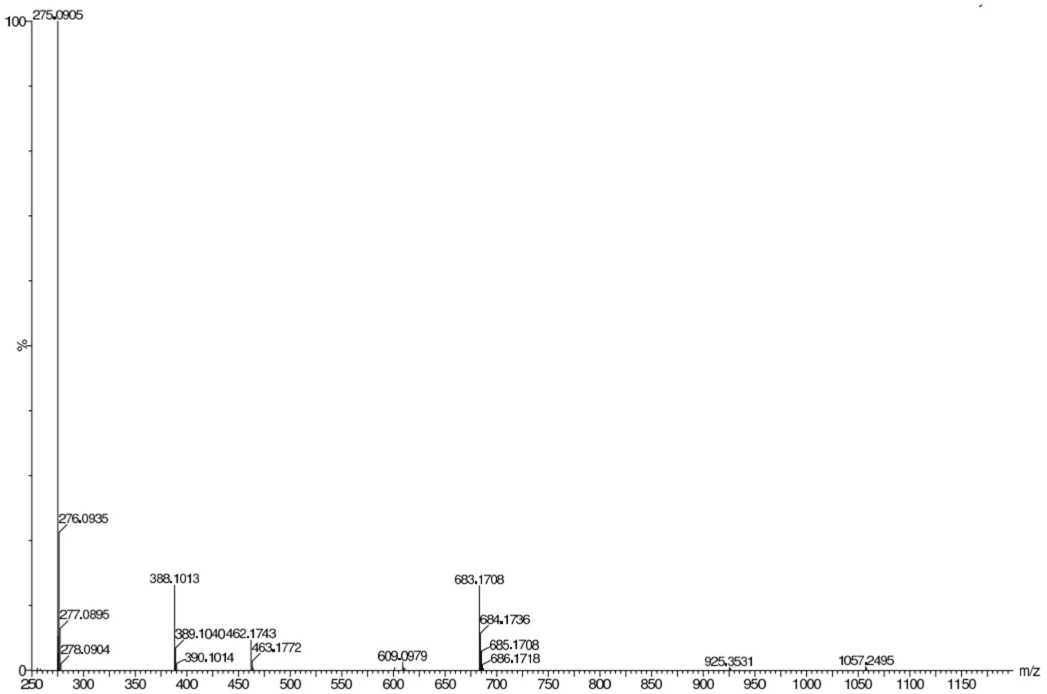

2.10. tert-butyl (1-oxo-1-((4'-(trifluoromethyl)-[1,1'-biphenyl])-3-sulfonamido)-3 (R)-(tritylthio)propan-2-yl)carbamate (6e)

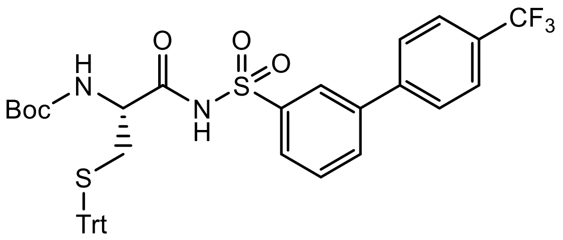

General procedure B was followed using **5e** (251 mg, 0.83 mmol), N-(tert-butoxycarbonyl)-S-tritylcysteine (459 mg, 1 mmol), HBTU (472 mg, 1.24 mmol) and triethylamine (366 µL, 2.47 mmol). The crude is purified using silica gel chromatography (30% EtOAc in heptane) to obtain the title compound **6e** as a yellow powder (167 mg, 27% yield).

^{1}H NMR (300 MHz, (MeOD): δ (ppm) = 8.30 (s, 1H), 8.02 (d, J= 8.90 Hz, 1H), 7.92 (d, J= 7.64 Hz, 1H), 7.77 (q, J= 8.44 Hz, 4H), 7.61 (t, J= 8.04 Hz, 1H), 3.93 (T, J= 6.91 Hz, 1H), 2.30 (d, J= 6.91 Hz, 2H), 1.35 (s, 9H) ^{13}C NMR (75 MHz, (MeOD): δ (ppm) = 144.24, 131.34, 129.99, 129.49, 128.81, 128.14, 127.68, 126.70, 126.52, 126.28, 56.22, 55.81, 25.38 HRMS (ESI): calcd. for $C_{40}H_{36}F_3N_2O_5S_2$ $[M-H]^-$: 745.2023, found: 745.2020.

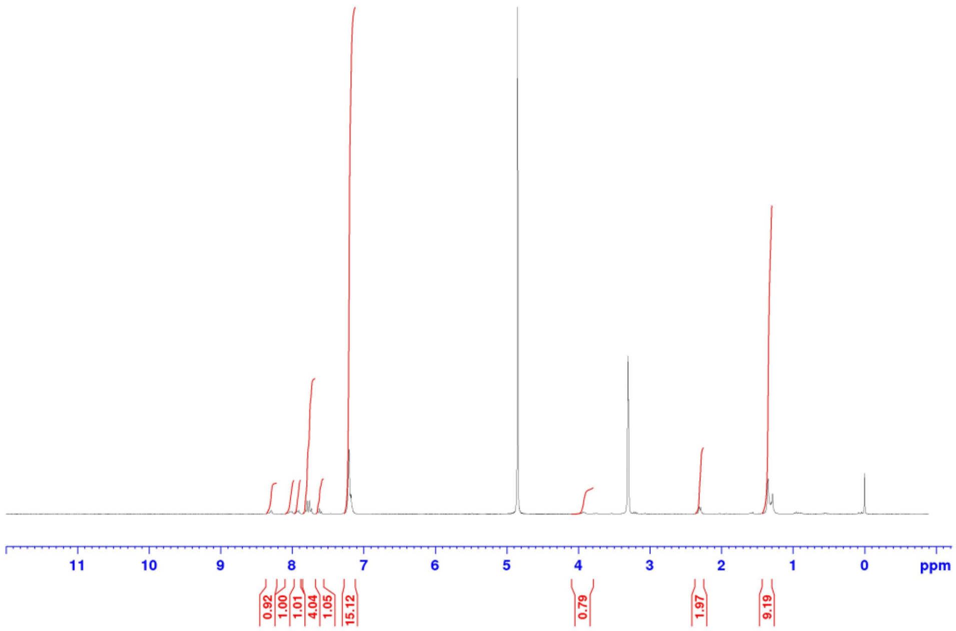

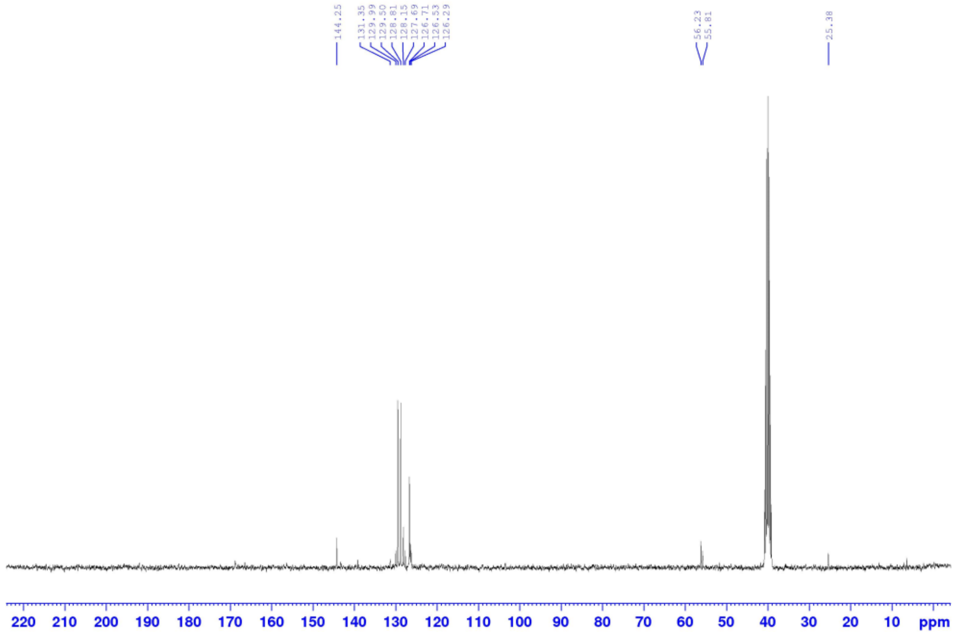

2.11. N-([1,1'-biphenyl]-3-ylsulfonyl)- 2 (R)-amino-3-mercaptopropanamide (7a)

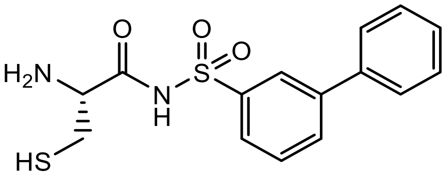

General procedure C was followed using **6a** (149 mg, 0.21 mmol) and triethylsilane (80 µL, 0.5 mmol). The resulting crude is purified using silica gel chromatography (5% MeOH in EtOAc) to obtain the final product **7a** (38 mg, 54 % yield).

^{1}H NMR (300 MHz, (DMSO): δ (ppm) = 8.12 (s, 1H), 7.99 (bs, 2H, NH), 7.85 (d, J= 7.53 Hz, 2H), 7.68 (s, 1H), 7.66 (s, 1H), 7.60 (t, J= 7.06 Hz, 1H), 7.51 (t, J= 7.29 Hz, 2H), 7.43 (d, J= 7.53 Hz, 1H), 3.83 (s, 1H), 2.91 (s, 2H); ^{13}C NMR (150 MHz, (DMSO): δ (ppm) = 129.23, 129.16, 129.10, 128.42, 128.13, 126.85, 126.32, 126.19, 125.53, 55.64, 25.21; HRMS (ESI): calcd. for $C_{15}H_{17}N_2O_3S_2$ $[M+H]^+$: 337.0675, found: 337.0678.

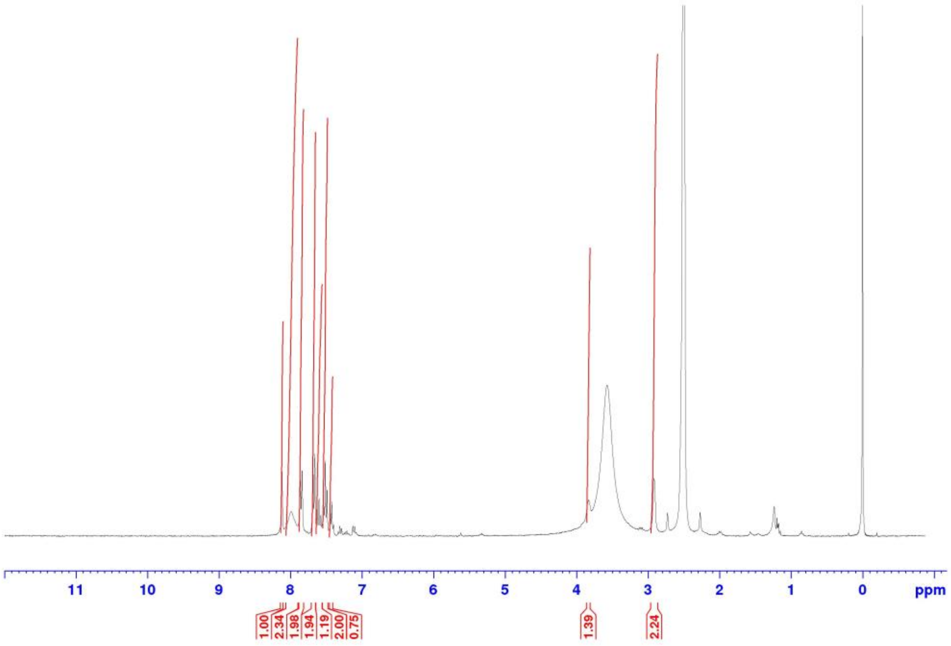

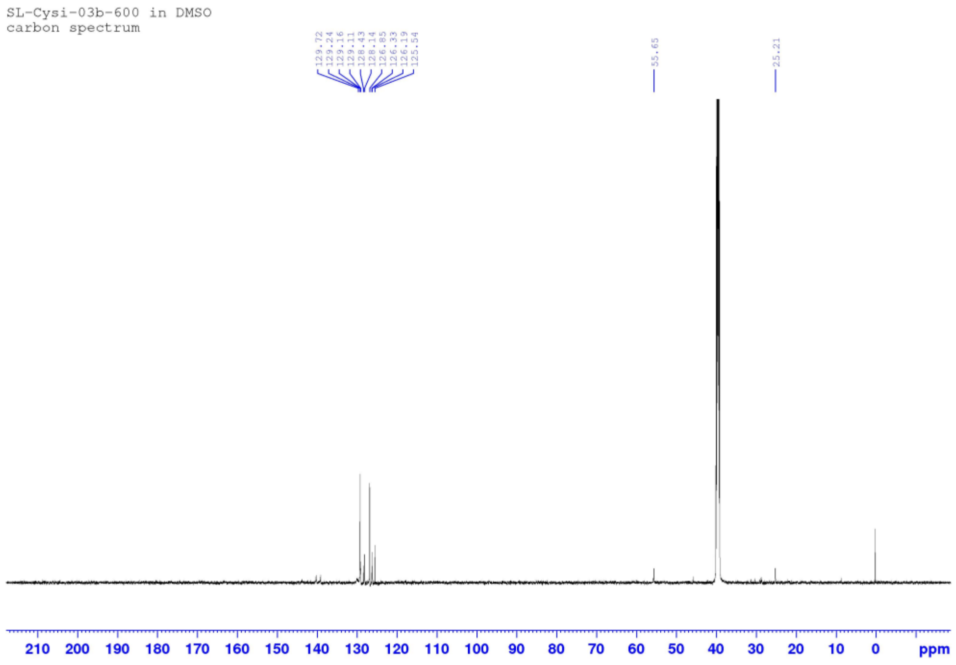

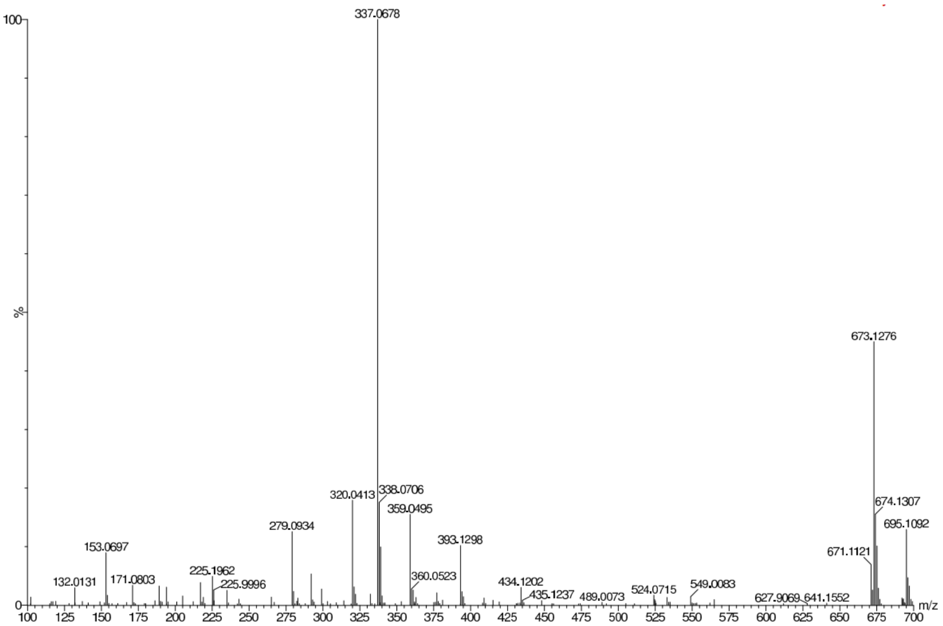

2.12. 2 (R)-amino-3-mercapto-N-((3-(6-methoxypyridin-3-yl)phenyl)sulfonyl)propenamide (7b)

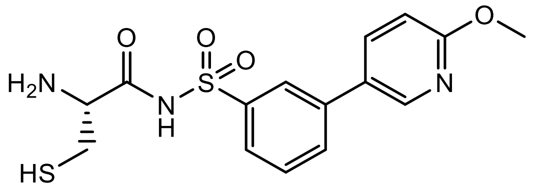

General procedure C was followed using **6b** (163 mg, 0.22 mmol) and triethylsilane (80 µL, 0.5 mmol). The resulting crude is purified using silica gel chromatography (5% MeOH in EtOAc) to obtain the final product **7b** (40 mg, 51 % yield).

^{1}H NMR (300 MHz, (DMSO): δ (ppm) = 8.50 (d, J= 2.22 Hz, 1H), 8.11-7.98 (m, 4H), 7.92-7.83 (m, 2H), 7.63 (t, J= 8.32 Hz, 1H), 6.95 (d, J= 7.21, 1H), 3.91 (s, 3H), 3.79 (s, 1H), 2.93-2.84 (m, 2H); ^{13}C NMR (150 MHz, (DMSO): δ (ppm) = 163,.53, 145.02, 137.73, 137.40, 129.37, 126.14, 124.96, 110.91, 53.47, 40.13, 30.40; HRMS (ESI): calcd. for $C_{15}H_{18}N_3O_4S_2$ [M+H]$^+$: 368.0733, found: 368.0730.

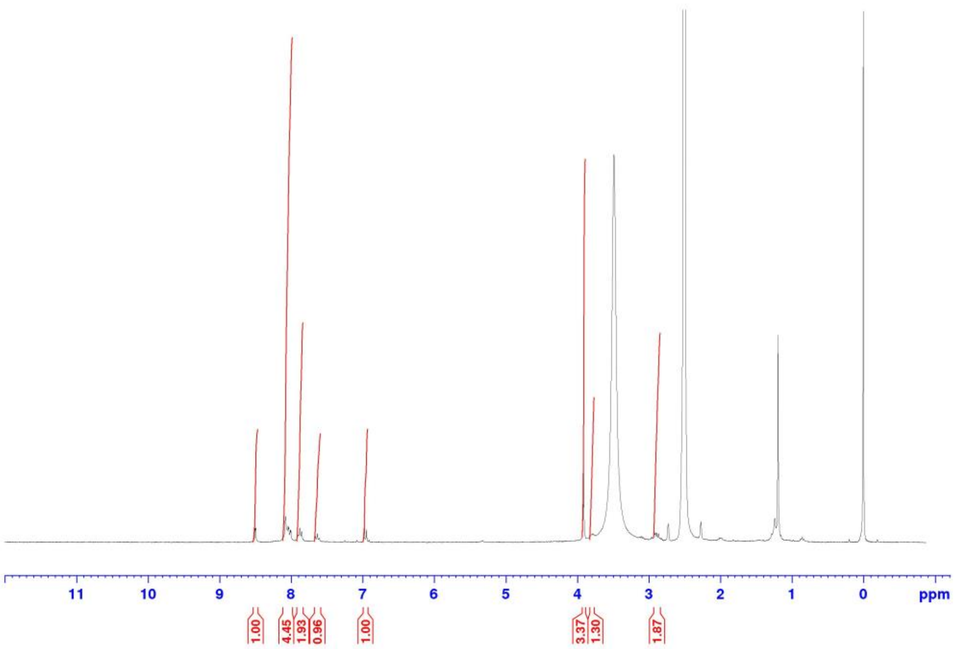
11
10
9
8
7
6
5
4
3
2
1
0
ppm
1.00
4.45
1.93
0.96
1.00
3.37
1.30
1.87

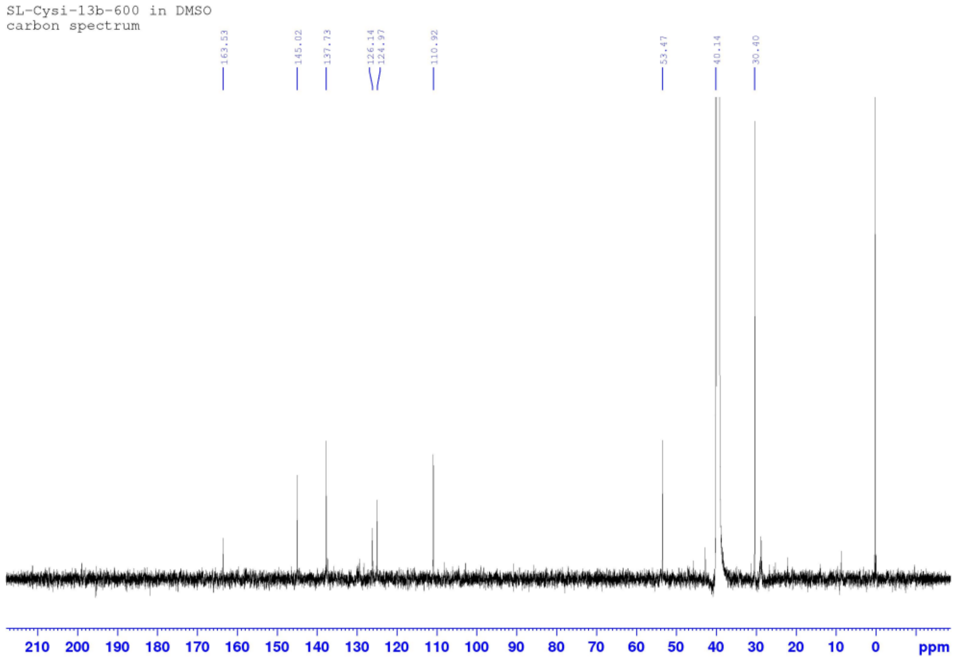
SL-Cysi-13b-600 in DMSO
carbon spectrum
163.53
145.02
137.73
126.14
124.97
110.92
53.47
40.14
30.40
210
200
190
180
170
160
150
140
130
120
110
100
90
80
70
60
50
40
30
20
10
0
ppm

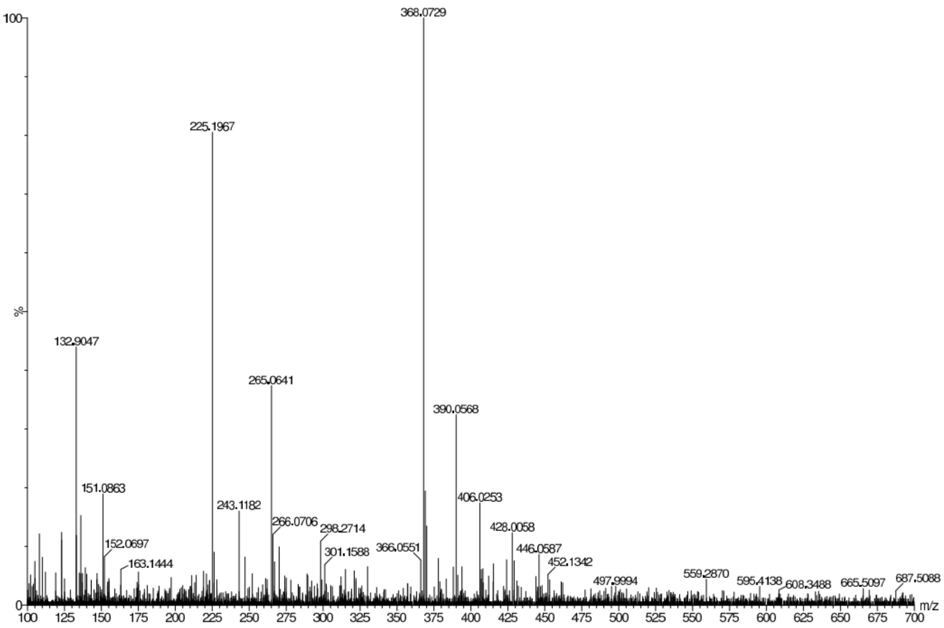

2.13. 2 (R)-amino-3-mercapto-N-((3-(pyridin-3-yl)phenyl)sulfonyl)propanamide (7c)

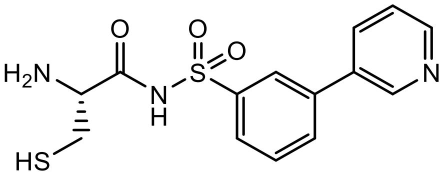

General procedure C was followed using **6c** (152 mg, 0.22 mmol) and triethylsilane (80 µL, 0.5 mmol). The resulting crude is purified using silica gel chromatography (5% MeOH in EtOAc) to obtain the final product **7c** (17 mg, 23 % yield).

^{1}H NMR (300 MHz, (DMSO): δ (ppm) = 8.97 (s, 1H), 8.70 (d, J= 4.54 Hz, 1H), 8.25 (d, J= 8.17 Hz, 1H, 8.18 (s, 1H), 8.05 (bs, 2H, NH), 7.97 (t, J= 8.17 Hz, 2H), 7.74-7.62 (m, 2H), 2.93 (s, 2H); ^{13}C NMR (75 MHz, (DMSO): δ (ppm) = 168.62, 140.56, 135.82, 130.43, 129.60, 129.00, 128.23, 126.69, 126.19, 125.04, 122.54, 55.90, 25.30

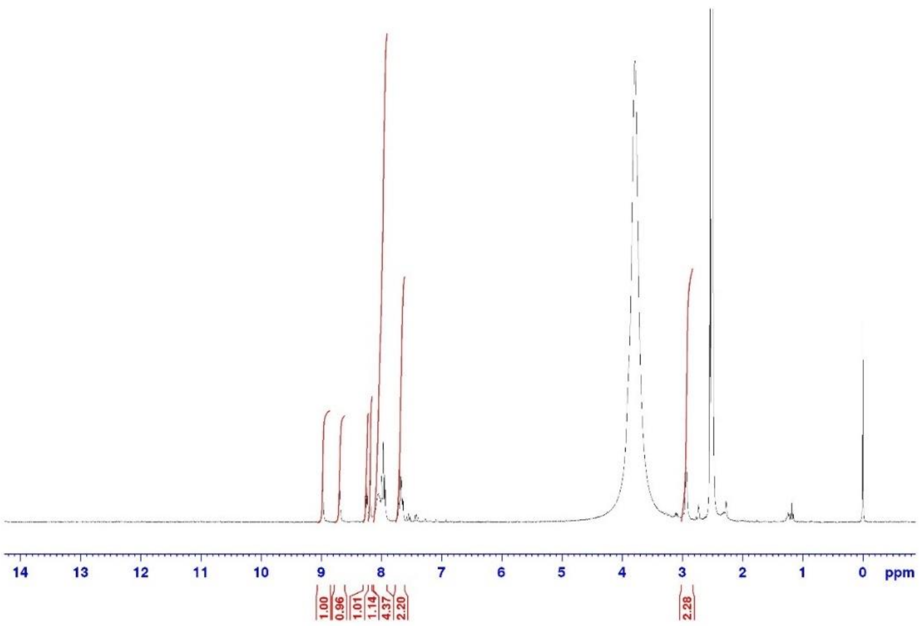

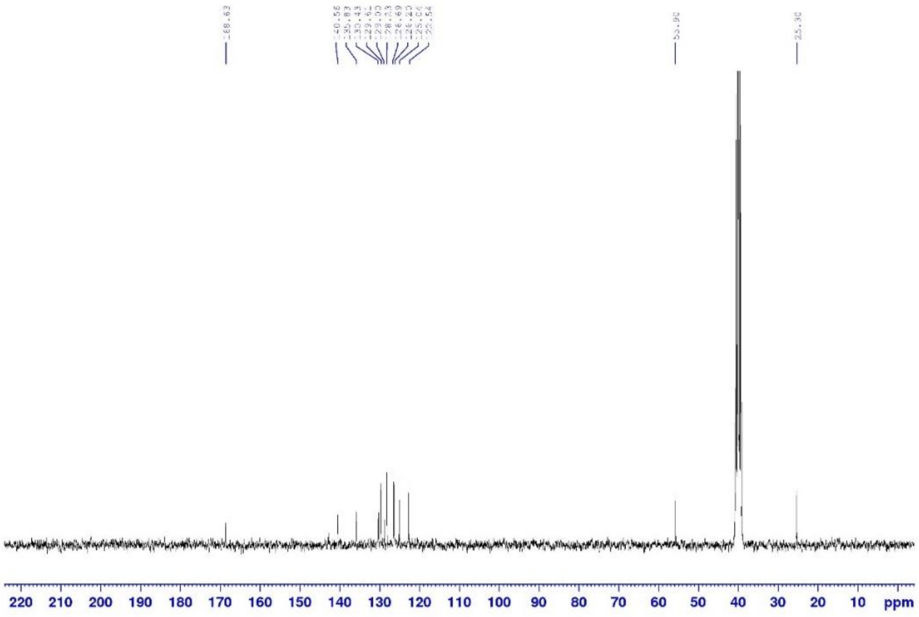

2.14. 2 (R)-amino-3-mercapto-N-((3-(thiophen-2-yl)phenyl)sulfonyl)propanamide (7d)

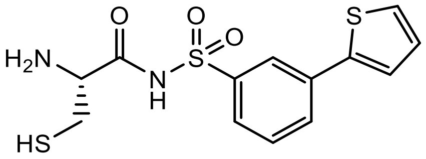

General procedure C was followed using **6d** (213 mg, 0.31 mmol) and triethylsilane (120 µL, 0.75 mmol). The resulting crude is purified using silica gel chromatography (5% MeOH in EtOAc) to obtain the final product **7d** (65 mg, 61 % yield).

^{1}H NMR (300 MHz, (DMSO): δ (ppm) = 8.13 (s, 1H), 8.05 (bs, 2H, NH), 7.98-7.89 (m, 2H), 7.80 (d, J= 8.26 Hz), 7.72-7.68 (m, 1H), 7.61-7.52 (m, 2H), 3.89 (s, 1H), 2.92 (d, J= 3.88 Hz, 2H); ^{13}C NMR (75 MHz, (DMSO): δ (ppm) = 130.19, 129.65, 128.17, 126.47, 126.26, 125.09, 122.68, 55.84, 25.42; MS (ESI): calcd. for $C_{13}H_{14}N_2O_3S_3$ $[M+H]^+$: 343.0, found: 342.8

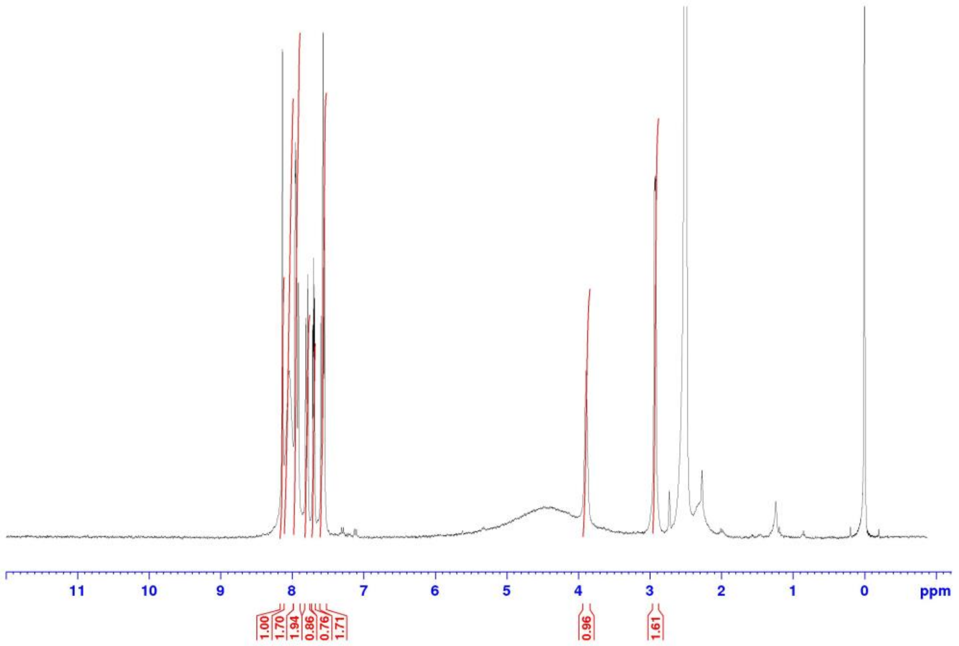

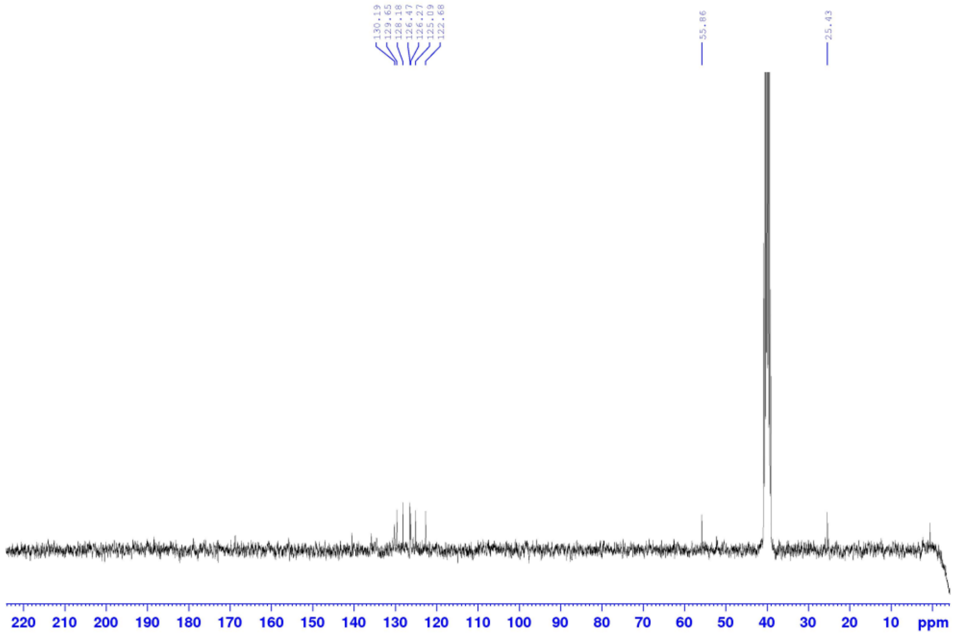

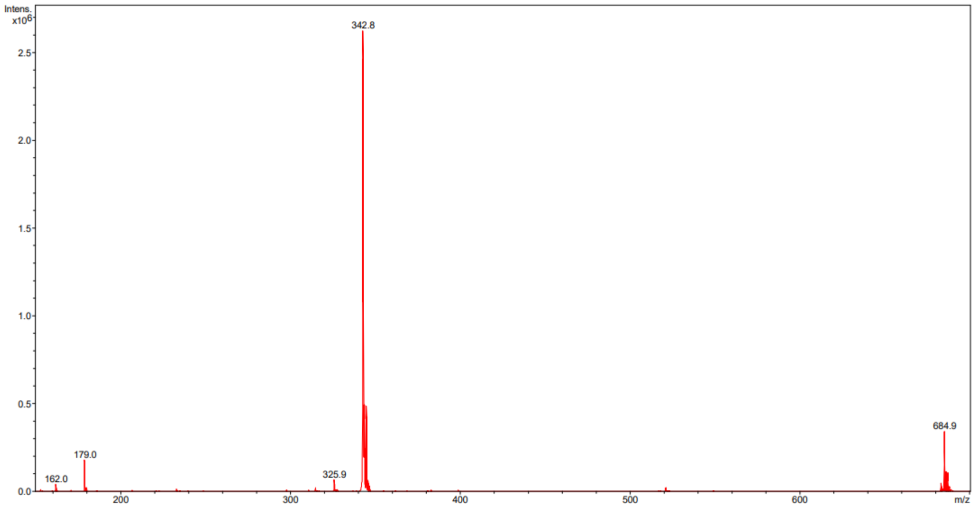

2.15. 2 (R)-amino-3-mercapto-N-((4'-(trifluoromethyl)-[1,1'-biphenyl]-3-yl)sulfonyl)propanamide (7e)

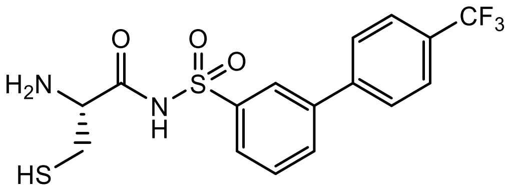

General procedure C was followed using **6e** (167 mg, 0.22 mmol) and triethylsilane (80 µL, 0.5 mmol). The resulting crude is purified using silica gel chromatography (5% MeOH in EtOAc) to obtain the final product **7e** (35 mg, 39 % yield).

^{1}H NMR (300 MHz, (DMSO): δ (ppm) = 8.17 (s, 1H), 8.00-7.83 (m, 8H), 7.64 (t, J= 7.30 Hz, 1H), 3.80 (s, 1H) 2.91 (s, 2H); ^{13}C NMR (150 MHz, (DMSO): δ (ppm) = 143.86, 19.11, 128.43, 127.70, 126.32, 126.07, 126.04, 125.80, 55.83, 29.05; HRMS (ESI): calcd. for $C_{16}H_{16}F_3N_2O_3S_2$ $[M+H]^+$: 405.1, found: 404.9

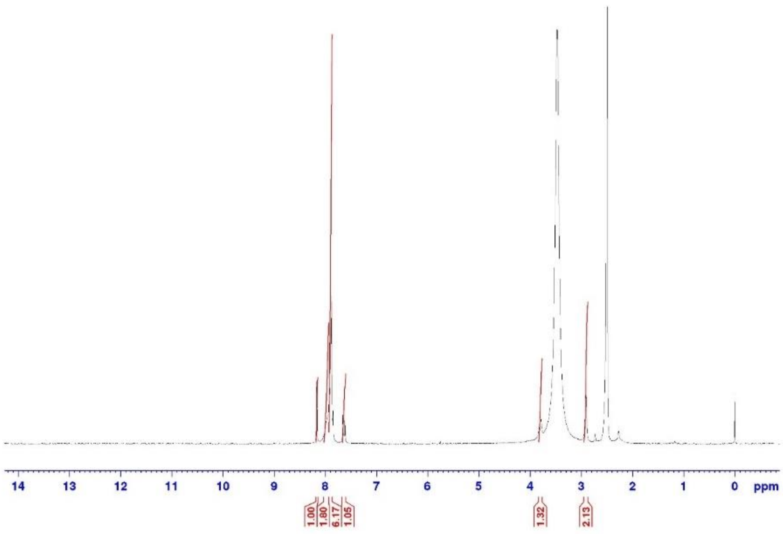

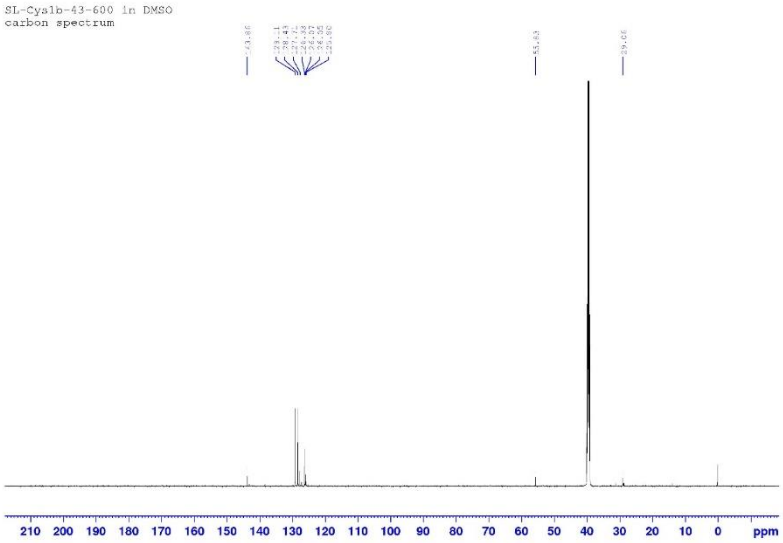
SL-Cys1b-43-600 in DMSO
carbon spectrum
55.83
210 200 190 180 170 160 150 140 130 120 110 100 90 80 70 60 50 40 30 20 10 0
ppm

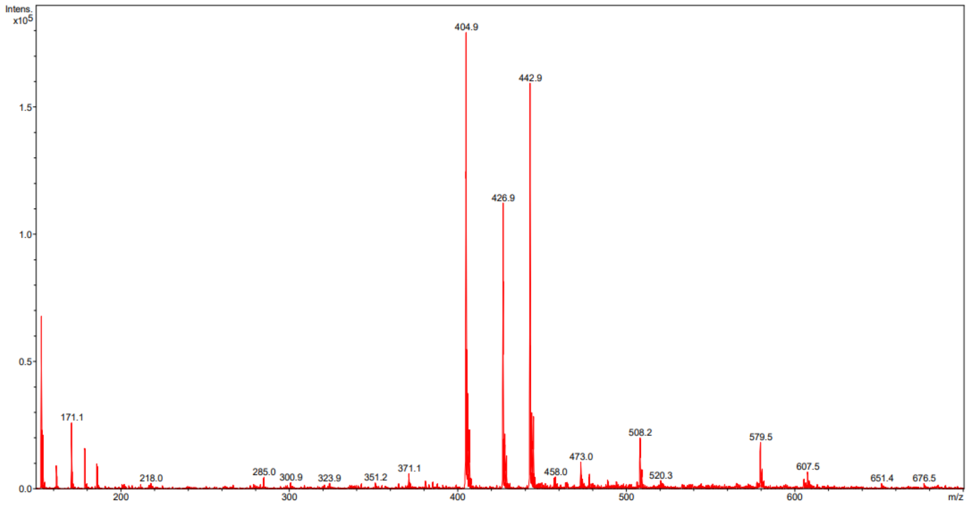
Intens.
x10^5
1.5
1.0
0.5
0.0
404.9
442.9
426.9
171.1
218.0
285.0
300.9
323.9
351.2
371.1
458.0
473.0
508.2
520.3
579.5
607.5
651.4
676.5
200
300
400
500
600
m/z
